# Supplementary material for: Autocatalytic selection of gene functions in synthetic cells
Source: Commun Biol. 2026 May 27;9:1124. doi: 10.1038/s42003-026-10372-z (PMC13493933; doi:10.1038/s42003-026-10372-z)
Supplement: Supplementary file 1 [file 42003_2026_10372_MOESM1_ESM.pdf]

nature portfolio

Peer Review File

Autocatalytic selection of gene functions in synthetic cells

Corresponding Author: Professor Christophe Danelon

This manuscript has been previously submitted at another journal. This document only contains information relating to versions considered at Communications Biology.

This file contains all reviewer reports in order by version, followed by all author rebuttals in order by version.

Version 0:

Reviewer comments:

Reviewer #1

(Remarks to the Author)
The work by Heras and Danelon describes autocatalytic selection (ACS) for transcription-translation coupled DNA replicators. ACS are general scheme that links the activity of a gene of interest (GOI) to the self-replication of a minimal DNA replicator (in this case based on the phage Phi29 genes) in compartments (e.g.liposomes), thereby enriching functional variants. The authors demonstrate three coupling modes (i) transcriptional (T7 RNAP encoded on the replicator), (ii) metabolic/regulatory (β-galactosidase converts lactose to allolactose to relieve LacI repression of Phi29 genes), and (iii) replication-level (a self-encoded Gmk supplies dGTP to drive DNA synthesis). Together, these results position ACS as a potentially modular route to evolve diverse functions in vitro and to integrate modules on the path to synthetic cells. The experiments are carefully executed and the core concept is timely and impactful for the SynBio-community.

1. Concepts similar to ACS exists already since a while for RNA-based genetic systems. Please mention them in your intro and / or discussion. https://journals.plos.org/plosgenetics/article?id=10.1371/journal.pgen.1010471 https://www.nature.com/articles/s41559-018-0650-z

2. Similarly, the authors fail to cite the ACS-equivalent multicomponent autocatalytic coupling of both tRNAs (https://pubs.acs.org/doi/full/10.1021/acssynbio.2c00163) and aminoacyl-tRNA synthetases (https://pubs.acs.org/doi/full/10.1021/acssynbio.3c00014) in self-encoded (PCR-free!) DNA replication in PURE. This creates the false impression for readers outside the field that the ACS concept is completely new, as implied in the third paragraph on page 2.

3. The work hinges on a modified PUREfrex formulation after the authors identified that the original formulation was changed and that Mg^{2+}/NTP is critical for replication. This is well documented and I appreciate the effort. Would it be possible to specify tolerance ranges (e.g., acceptable windows for Mg^{2+} and each NTP) and report performance across ≥2 independent reagent batches. This would be very valuable for the community as a whole.

3. On page 7, line 214, the authors write: "Under a non-limiting T7 RNAP regime in liposomes, the amplification folds for ori-tag-p2p3 and ori-T7-p2p3 were 5.3 and 4.4, respectively. This subtle enrichment of the ori-tag-p2p3 template aligns with observations from individual template experiments, where ori-tag-p2p3 replicated slightly better than ori-T7-p2p32 when sufficient T7 RNAP was available (Fig. 3a)." Looking at the data, in particular in Figure 3d, I doubt that this difference in enrichment is significant. Also for the comparison / discussion of the other results in Figure 3 under limiting T7-RNAP conditions, t-tests would be very helpful to avoid over-interpretation of subtle differences. Generally, also error propagation should be performed for all the fold enrichments in the manuscript to improve scientific rigour.

4. Under limiting T7 RNAP in liposomes, ori-T7-p2p3 achieves 2.7x amplification vs 1.6x for the control (enrichment roughly 1.7x), whereas in bulk the shorter control strongly dominates (enrichment 6.8x). These are convincing as proof-of-principle but may be marginal for iterative evolution experiments. Have the authors considered further lowering input DNA to reduce co-encapsulation, or tightening selection (e.g. via further reducing exogenous T7 RNAP and extending incubation)?

5. In the co-encapsulation experiments on page 12, 250 nM of DNA was used in the 1:1 experiments and 100 nM vs. 400 nM of the active and inactive variants, respectively, in the enrichment experiments. It would be helpful to list the report λ and

expected co-encapsulation probabilities under both conditions (but also other instances of co-encapsulation) and put the results into context.

Minor comments:

- Does the heat-inactivation of DNAse I compromise liposome integrity?

- The “inactive” lacZ_E538Q mutant construct still shows some activity based on ref 19. A quick back-of-the-envelope calculation assuming typical sub microM expression levels of LacZ suggests that micromolar amounts of allolactose might produced during incubation. Those might be sufficient to inactivate LacI (Kd = 600 nM) and explain some of the background depression.

- In the abstract and main text, the authors use the wording “synthesis“ of a deoxynucleoside triphosphate. This wording can be interpreted as de-novo nucleotide biosynthesis. Since the GOI enables Gmk-mediated conversion of dGMP to dGTP (with NDP kinase providing the second phosphorylation) in the PURE system, this is more precisely in situ activation/regeneration via salvage triphosphorylation rather than de-novo synthesis. I suggested to edit the abstract:, e.g. “ACS of three functions: transcription, in situ triphosphorylation that regenerates dGTP from dGMP to support DNA replication, and β-galactosidase activity.” for reasons of clarity.

Reviewer #2

(Remarks to the Author)
This manuscript presents a proof-of-concept (PoC) study in which the authors attempt to apply an integrated DNA replication–transcription–translation system (IVTTR), based on their previously developed phi29 DNA replication machinery combined with the cell-free PURE system, to the evolution of genes using a liposome-based platform. As genes of interest (GOIs) for validation, they first examined the T7 RNA polymerase and demonstrated that autocatalytic selection is observed when the concentration of T7 RNA polymerase in the PURE system is extremely low. Next, they selected beta-galactosidase as a GOI and found that selection occurs when LacI and its regulatory factor, lactose, are introduced to link the DNA replication system to expression. Finally, they targeted Gmk, which participates in the pathway converting dGMP to dGTP, and attempted a similar experiment. They found that substrate exchange between liposomes posed a bottleneck, but reported that the introduction of cholesterol into the liposome membrane partially suppressed this issue.

Similar studies include the group’s own previous report in 2024 (Ref. 5), as well as the work of Okauchi and Ichihashi (Continuous cell-free replication and evolution of artificial genomic DNA in a compartmentalized gene expression system, ACS Synth Biol, 2021). Both studies reported continuous evolution of DNA polymerases using similar liposome-based systems. The latter paper has not been cited in this manuscript; it should be cited, and the Introduction should be revised accordingly. At present, the positioning of this work within and beyond the field is not clearly articulated.

Unlike those earlier studies, which actually tested the evolution of proteins, the present work only presents PoC experiments for GOIs that are not directly involved in replication but only indirectly connected. Therefore, in terms of novelty and significance, this manuscript falls somewhat short. If continuous evolution exploiting the high mutation rate of replication enzymes, or at least selection based on random mutagenesis, were demonstrated as in the previous reports, the results would be more suitable for publication in this journal.

Major Comment
- Data are presented suggesting that selection occurs even under conditions where multiple DNA molecules are present in a single liposome. As the authors themselves note in the Discussion, under the conditions of giant liposomes at 250 pM DNA concentration, co-encapsulation of two DNA species is expected. Nevertheless, as shown in Fig. 3c, the two DNA templates exhibit different amplification efficiencies, which may represent novel findings. However, in Fig. 5a, the figure appears to imply single-copy DNA encapsulation, making it unclear whether the experiments were actually performed at the single-copy level or not. This point needs to be clarified. If all experiments were performed at 250 pM DNA concentration, then the fact that DNA enrichment occurs under such conditions could indeed constitute a novel insight. Investigating the DNA concentration threshold at which selection occurs may provide an opportunity to present new and valuable findings.

Minor Comments
Abstract
- References should not be included in the abstract.
- The claim that beta-galactosidase is 'unrelated to the Central Dogma' is misleading. Since beta-galactosidase functions as part of the Lac operator system also used in this manuscript, it is in fact closely related to the Central Dogma.

Introduction
- The paragraph in lines 23–37 should objectively describe the positioning of this work within the field. Instead, it begins with detailed descriptions of the authors’ prior studies, giving the impression of a narrow, incremental development. It would be more effective to start from a broader perspective and emphasize the importance and novelty of this work in the context of the field.

Results
- The order of genes differs between figures: p2–p3–GOI in Fig. 1 (conceptual scheme), GOI–p2–p3 in Fig. 2 (T7 RNAP experiment), p2–p3–GOI in Fig. 4 (lacZ experiment), and GOI–p2–p3 in Fig. 6 (Gmk experiment). If there is a rationale for these different arrangements, it should be described; otherwise, it should be explicitly stated that there is no particular reason.

Version 1:

Reviewer comments:

Reviewer #2

(Remarks to the Author)
First, I would like to note that the overall positioning of the study now appears to be clearer, and I appreciate that the authors have addressed my previous comments sincerely, including performing additional experiments.

However, I would like to raise one remaining concern regarding the authors' reply.

The authors state:
"We want to emphasize that - unlike earlier studies - the selectable gene lacZ and activity of its encoded enzyme beta-galactosidase are NOT directly involved in replication nor in transcription and translation. This is precisely the most impactful and innovative aspect of our ACS scheme as potentially any GOI can be selected through the implementation of a biomolecular circuit coupled to the central dogma."

It is true that beta-galactosidase is not directly involved in the processes of the central dogma. However, my previous comment pointed out that it is indirectly involved. When I first read the manuscript, the activity of beta-galactosidase initially appeared unrelated to the central dogma, and I was therefore curious to see what strategy the authors had used to achieve this. However, it eventually became clear that the reaction product generated by the beta-galactosidase simply perturbs the DNA binding of a transcriptional regulator. Thus, the system is still closely linked to the central dogma.

In their response, the authors claim that any GOI can be selected, but it seems that the system still requires the GOI to be connected to the central dogma at some step. Therefore, I consider the authors' statement is overstated.

The authors also state:
"We disagree; beta-galactosidase is not a universal component for the flow of genetic information (central dogma). We designed a genetic circuit to couple the enzymatic activity to DNA replication. The evolvable function is the conversion of lactose into allolactose (it could virtually be any other reaction), while the introduction of the lac operator site links this function to the central dogma for selection."

My previous comment did not claim that beta-galactosidase is a universal component of the central dogma. Rather, my point was that it is connected to the processes of the central dogma, and therefore the claim that it is 'unrelated' is misleading. I do not understand why the authors have interpreted my comment in this way and continue to maintain this claim.

The claims of a paper are among its most important elements and must be phrased in a way that avoids misunderstanding. In particular, as a reviewer, I cannot accept a situation in which an explicit claim - especially one stated in the Abstract - contains misleading wording. Since it is clear that beta-galactosidase in this system is not unrelated to the processes of the central dogma, I think that at least the word 'unrelated' should not be used in the Abstract.

Open Access This Peer Review File is licensed under a Creative Commons Attribution 4.0 International License, which permits use, sharing, adaptation, distribution and reproduction in any medium or format, as long as you give appropriate credit to the original author(s) and the source, provide a link to the Creative Commons license, and indicate if changes were made.
In cases where reviewers are anonymous, credit should be given to 'Anonymous Referee' and the source.
The images or other third party material in this Peer Review File are included in the article's Creative Commons license, unless indicated otherwise in a credit line to the material. If material is not included in the article's Creative Commons license and your intended use is not permitted by statutory regulation or exceeds the permitted use, you will need to obtain permission directly from the copyright holder.
To view a copy of this license, visit https://creativecommons.org/licenses/by/4.0/

Subject: Revision for Manuscript **COMMSBIO-25-7564-T**
Title: Autocatalytic selection of gene functions in vitro
Authors: Laura Sierra Heras and Christophe Danelon

Dear Editorial Team,

We thank the two reviewers for their constructive comments that helped us improve the manuscript. The Referee's report is in **black text** and our point-by-point response is in **blue text**. Changes in the manuscript (main text and Supplementary information) are highlighted in **red text**.

We are pleased to provide you with a substantially revised manuscript that addresses all referees' concerns. In particular:

- We modified the Introduction to better position our work with regard to other studies involving a similar concept of autocatalytic selection in RNA-based and DNA-based genetic systems. An overview of previously described mechanisms is presented in the new Supplementary Table 1.
- We performed statistical tests to provide a more rigorous comparison of amplification fold differences.
- We fully engaged with the guidance from reviewer #2 and performed additional experiments for the selection of a metabolically active replicator starting from an NNS-degenerate library of the *lacZ* E538 codon.

+++++

Reviewers' comments:

Reviewer #1 (Remarks to the Author):

The work by Heras and Danelon describes autocatalytic selection (ACS) for transcription-translation coupled DNA replicators. ACS are general scheme that links the activity of a gene of interest (GOI) to the self-replication of a minimal DNA replicator (in this case based on the phage Phi29 genes) in compartments (e.g.liposomes), thereby enriching functional variants. The authors demonstrate three coupling modes (i) transcriptional (T7 RNAP encoded on the replicator), (ii) metabolic/regulatory (β-galactosidase converts lactose to allolactose to relieve LacI repression of Phi29 genes), and (iii) replication-level (a self-encoded Gmk supplies dGTP to drive DNA synthesis). Together, these results position ACS as a potentially modular route to evolve diverse functions in vitro and to integrate modules on the path to synthetic cells. The experiments are carefully executed and the core concept is timely and impactful for the SynBio-community.
Reply: We thank the reviewer for acknowledging the importance of our work and for the valuable suggestions.

1. Concepts similar to ACS exists already since a while for RNA-based genetic systems. Please

mention them in your intro and / or discussion.
https://journals.plos.org/plosgenetics/article?id=10.1371/journal.pgen.1010471 
https://www.nature.com/articles/s41559-018-0650-z

Reply: We agree in general (see also next point) that our work needs better positioning within the field. We initially limited our discussion of the ACS concept to DNA-based systems. Note that the work of Mizuuchi and Ichihashi was cited as ref. 7.

In the revised manuscript, we gave a broader perspective by extending the introduction to RNA-based genetic systems as a possible earlier type of ACS involving nucleic acids before the emergence of self-replicating DNA systems. Specifically, we added:
"Artificial RNA-based replicating systems coupled to translation have been shown to undergo long-term evolution in vitro[2]."

"In previous studies, a similar concept was applied to an RNA-based genetic system that coupled RNA replication to a metabolic activity, resulting in Darwinian selection driven by the encoded enzymatic function[6,10]."

We motivated our choice to use a DNA-based system in the present study: "While RNA-based systems offer powerful models for early evolutionary processes, DNA-based self-replicators provide increased genetic stability and information capacity, making them more suitable for the long-term integration and evolution of the multiple functional modules required for a synthetic cell."

Please, see next point for further changes in the Introduction.

2. Similarly, the authors fail to cite the ACS-equivalent multicomponent autocatalytic coupling of both tRNAs (https://pubs.acs.org/doi/full/10.1021/acssynbio.2c00163) and aminoacyl-tRNA synthetases (https://pubs.acs.org/doi/full/10.1021/acssynbio.3c00014) in self-encoded (PCR-free!) DNA replication in PURE. This creates the false impression for readers outside the field that the ACS concept is completely new, as implied in the third paragraph on page 2.
Reply: The introduction has been revised accordingly. For instance, we wrote:
"Furthermore, autocatalytic coupling between the expression of transfer RNAs[18], aminoacyl-tRNA synthetases[19] or dNTP regeneration[20] and self-encoded DNA replication has been reported, but the genetic designs did not support integration of both functions on the same chromosome."

For completeness, we also made a comprehensive table (new Supplementary Table 1) that summarizes the most relevant studies presenting equivalent concepts of ACS of gene functions. To our knowledge, our work demonstrates for the first time that functional selection drives the integration of genes in a self-replicating DNA chromosome.

We also changed "in vitro" by "in synthetic cells" in the title to better differentiate our work from other studies.

3. The work hinges on a modified PUREfrex formulation after the authors identified that the original formulation was changed and that Mg^{2+}/NTP is critical for replication. This is well documented and I appreciate the effort. Would it be possible to specify tolerance ranges (e.g., acceptable windows for Mg^{2+} and each NTP) and report performance across ≥2 independent reagent batches. This would be very valuable for the community as a whole.
Reply: We thank the reviewer for recognizing the importance of the optimized PURE*frex* formulation in supporting DNA replication. In the present study, the composition used, referred to as M1, was selected based on the conditions reported by Seo and Ichihashi et al. [ref 23], who systematically examined the effect of Mg^{2+} and NTP concentrations for efficient replication in a transcription/translation-coupled DNA replication system (see Figure 7 in their paper). Although their system differs mechanistically from ours, we found that adopting their optimized Mg^{2+}/NTP balance also enabled replication in our setup. For comparison, we tested the standard PURE*frex* formulation and a modified composition but with standard Mg^{2+}/NTP concentrations (M2, Supplementary Fig. 1), which did not yield replication. Apart from these three compositions, we did not conduct an independent exploration of tolerance ranges for Mg^{2+} and NTP concentrations and used M1 consistently throughout the study. Notably, the work by Seo and Ichihashi et al. already provides a detailed analysis of Mg^{2+}/NTP parameter space, which we consider a valuable reference for further optimization.

Regarding batch reproducibility, all experiments reported here were performed using two independent reagent batches. As reagents were replenished individually when depleted, the experiments occasionally involved mixed batches of certain components. Therefore, a direct comparison between fully independent batches was not possible. Nonetheless, no qualitative differences in replication outcomes were observed across experiments, indicating that the results are robust under the reported conditions.
We also acknowledge that this information is valuable for the community and added a short text in Methods, subsection "Optimization of PURE composition for IVTTR reactions".

3. On page 7, line 214, the authors write: "Under a non-limiting T7 RNAP regime in liposomes, the amplification folds for ori-tag-p2p3 and ori-T7-p2p3 were 5.3 and 4.4, respectively. This subtle enrichment of the ori-tag-p2p3 template aligns with observations from individual template experiments, where ori-tag-p2p3 replicated slightly better than ori-T7-p2p32 when sufficient T7 RNAP was available (Fig. 3a)." Looking at the data, in particular in Figure 3d, I doubt that this difference in enrichment is significant. Also for the comparison / discussion of the other results in Figure 3 under limiting T7-RNAP conditions, t-tests would be very helpful to avoid over-interpretation of subtle differences. Generally, also error propagation should be performed for all the fold enrichments in the manuscript to improve scientific rigour.
Reply: We fully engaged with the reviewer's comment and performed statistical tests (two-tailed paired *t*-tests) of the data throughout the manuscript, not only in Figure 3.

Figure 3a reports results from two biological replicates. Running a statistical test with n=2 is not relevant. This experiment was designed to find the concentration of T7 RNAP that gives a high differential amplification between the two constructs before performing selection assays. The trend showing that under severing conditions for T7 RNAP, ori-T7-p2p3 is more efficiently replicated than ori-tag-p2p3 is very clear. Our main claim is not that ori-tag-p2p3 replicates better than ori-T7-p2p3 at high T7 RNAP concentration.

Figure 3c:

	**rep1**	**rep2**	**rep3**	**Two-tailed P value (paired *t* test)**	**Significance (P<0.05)**
**Liposome 1X**					
tag	4.765	5.6	5.53651235	0.14407068	n.s.
T7	3.413	5.4	4.36063834		
**Liposome 30X**					
tag	1.573	1.3	1.80716189	0.0338794	*
T7	2.883	2.0	3.1904827		
**Bulk 30X**					
tag	49.181	38.8	55.5321521	0.00919309	**
T7	7.644	4.0	6.84676036		

The difference between the mean values for the ori-tag-p2p3 and ori-T7-p2p3 in the condition Liposome 1X is not statistically significant. We therefore rephrased the sentence mentioned by the reviewer as: "Under a non-limiting T7 RNAP regime in liposomes, the amplification folds for ori-tag-p2p3 and ori-T7-p2p3 were similar (no statistically significant difference) ~~5.3 and 4.4, respectively. This subtle enrichment of the ori-tag-p2p3 template aligns with observations from individual template experiments, where ori-tag-p2p3 replicated slightly better than ori-T7-p2p32 when sufficient T7 RNAP was available (Fig. 3a)~~."
This does not change at all our conclusions.

Figure 4e:

	**rep1**	**rep2**	**rep3**		**Two-tailed P value (paired *t* test)**	**Significance (P<0.05)**
**1**						
**WT**	3.9742870626076	8.692	6.526		0.865345667	n.s.
**MUT**	4.6122647807363	5.976	7.880			
**2**						
**WT**	1.2418954654223	1.424	1.291		0.89496381	n.s.
**MUT**	1.4337273572314	1.224	1.248			
**3**						
**WT**	6.6409193989547	7.968	7.365		0.002811472	**
**MUT**	1.4334221461013	1.724	1.418			

Figure 5b:

	Rep1	Rep2	Rep3		Two-tailed P value (paired *t* test)	Significance (P<0.05)
- LacI - lactose						
t0	0.28	0.28	0.28		0.36906164	n.s.
t6	0.27	0.28	0.28			
+ LacI + lactose						
t0	0.29	0.31	0.29		0.00677412	**
t6	0.48	0.51	0.44			

Figure 6c:

	rep1	rep2	rep3	rep4	Two-tailed P value (paired *t* test)	Significance (P<0.05)
Population mix No cholesterol						
tag	8.533	5.112	6.181	10.132	0.02049	*
gmk	24.695	12.498	15.147	29.652		

In the revised manuscript, a 4th repeat was performed and included in the analysis.

Figure 6d:

	rep1	rep2	rep3	rep4	Two-tailed P value (paired *t* test)	Significance (P<0.05)
Population mix With cholesterol						
tag	1.720	1.471	1.891	2.216	0.02957	*
gmk	13.392	3.835	12.935	15.393		

In the revised manuscript, a 4th repeat was performed and included in the analysis.

Figure 6e:

	rep1	rep2	rep3	Two-tailed P value (paired *t* test)	Significance (P<0.05)
DNA mix					
tag	1.277	1.459	1.333	0.04076729	*
gmk	4.268	3.085	3.053		

About error propagation. We calculated enrichment from each replicate individually, then we computed the mean (bar height in Fig. 3d). Error propagation would have been needed if we had calculated (relative) enrichment from the individual qPCR data points, but this is not what we did. For clarity, we specified in the figure legend that enrichment was calculated for each replicate:

“Enrichments were derived independently from amplification fold values for each biological replicate. Source data are available for this figure.”

4. Under limiting T7 RNAP in liposomes, ori-T7-p2p3 achieves 2.7x amplification vs 1.6x for the control (enrichment roughly 1.7x), whereas in bulk the shorter control strongly dominates (enrichment 6.8x). These are convincing as proof-of-principle but may be marginal for iterative evolution experiments. Have the authors considered further lowering input DNA to reduce co-encapsulation, or tightening selection (e.g. via further reducing exogenous T7 RNAP and extending incubation)?
Reply:
About iterative evolution. In the original manuscript, we acknowledged that iterative cycles may be required when the amplification fold is not very strong: “*Iterative cycles may be required… selection stringency is preferred.*”

We would like to emphasize that ori-T7-p2p3 is a 6.1-kb construct, whereas the control ori-tag-p2p3 is only 3.3 kb. Despite this substantial size difference, ori-T7-p2p3 still achieves enrichment under limiting T7 RNAP conditions, which is a remarkable outcome given the intrinsic replication advantage of shorter templates observed in non-selective bulk reactions. Therefore, if the selection mechanism is sufficient to allow for significant enrichment of a construct twice the size of the competitor, even higher enrichment values can be anticipated when comparing constructs of similar size but differing in activity, as in iterative evolution experiments.
To demonstrate that selection of active variants is feasible even when the enrichment as measured by qPCR is not very high (<10), we performed IVTTR starting from a library of ori-p2p3-lacZ, where mutations were introduced to inactive β-galactosidase (new Fig. 5c,d and Fig. S12-13). After a single round, active variants were successfully enriched.

About tightening selection. As we suggested for the replication of ori-T7-p2p3, it is possible to further refine selection parameters—such as T7 RNAP and DNA concentrations, as well as incubation times—to maximize selection efficiency in each experiment.

About lowering input DNA concentration to reduce co-encapsulation. We performed experiments with 50 pM (instead of 500 pM, Fig. 3a) of ori-tag-p2p3 and ori-T7-p2p3 in separate liposome populations (figure below). At such a DNA concentration, the probability that two copies of DNA are co-encapsulated is lower (0.49 as calculated with the provided code, see Comment #5 below), further reducing the co-encapsulation probability of two DNA species in competition assays.

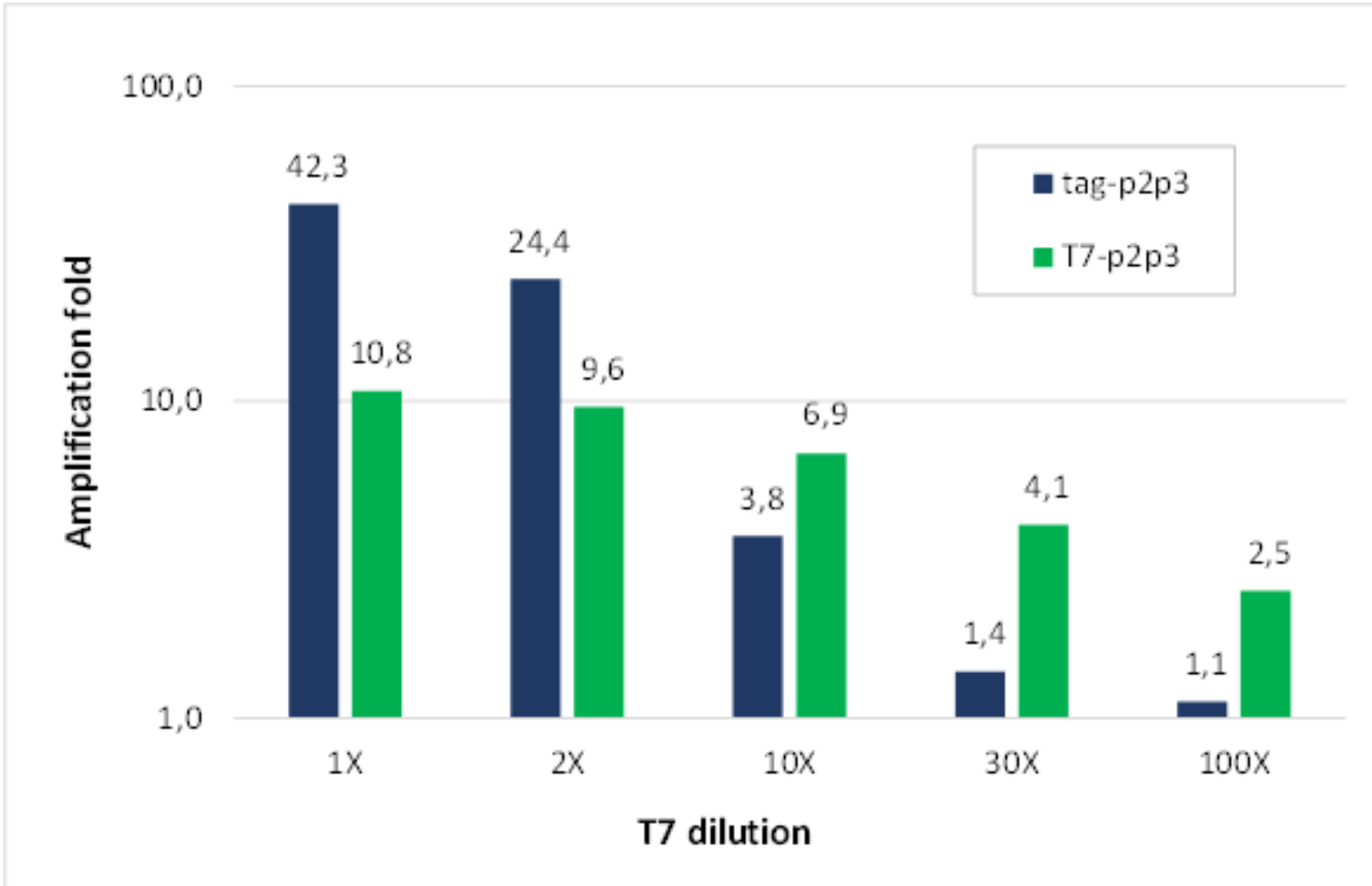

The trend of the amplification fold as a function of the concentration of purified T7 RNAP is similar as with 500 pM DNA (Fig. 3a): when purified T7 RNAP becomes limiting, replication is maintained only when T7 RNAP is regenerated from ori-T7-p2p3. Considering the tradeoffs for DNA concentration, selection pressure stringency and replication efficiency, we decided to use 500 pM total DNA and 30x diluted T7 RNAP for the competition assay with mixed DNA (Fig. 3b-d). Despite the higher probability of DNA co-encapsulation at 500 pM total DNA (250 pM each) than at 50 pM, we found a good compromise between expression and selective replication yielding enrichment of ori-T7-p2p3 over ori-tag-p2p3. The optimal DNA concentration and selection pressure conditions are case specific and should be determined for every system investigated.

5. In the co-encapsulation experiments on page 12, 250 nM of DNA was used in the 1:1 experiments and 100 nM vs. 400 nM of the active and inactive variants, respectively, in the enrichment experiments. It would be helpful to list the report λ and expected co-encapsulation probabilities under both conditions (but also other instances of co-encapsulation) and put the results into context.

Reply: We used 250 pM of DNA in the 1:1 experiments and 100 pM vs 400 pM in the enrichment experiments, not "nM".

We modified Supplementary Fig. 6 to also include simulations for the 1:4 condition and the case where only 10% of DNA is transcriptionally active (figure below):

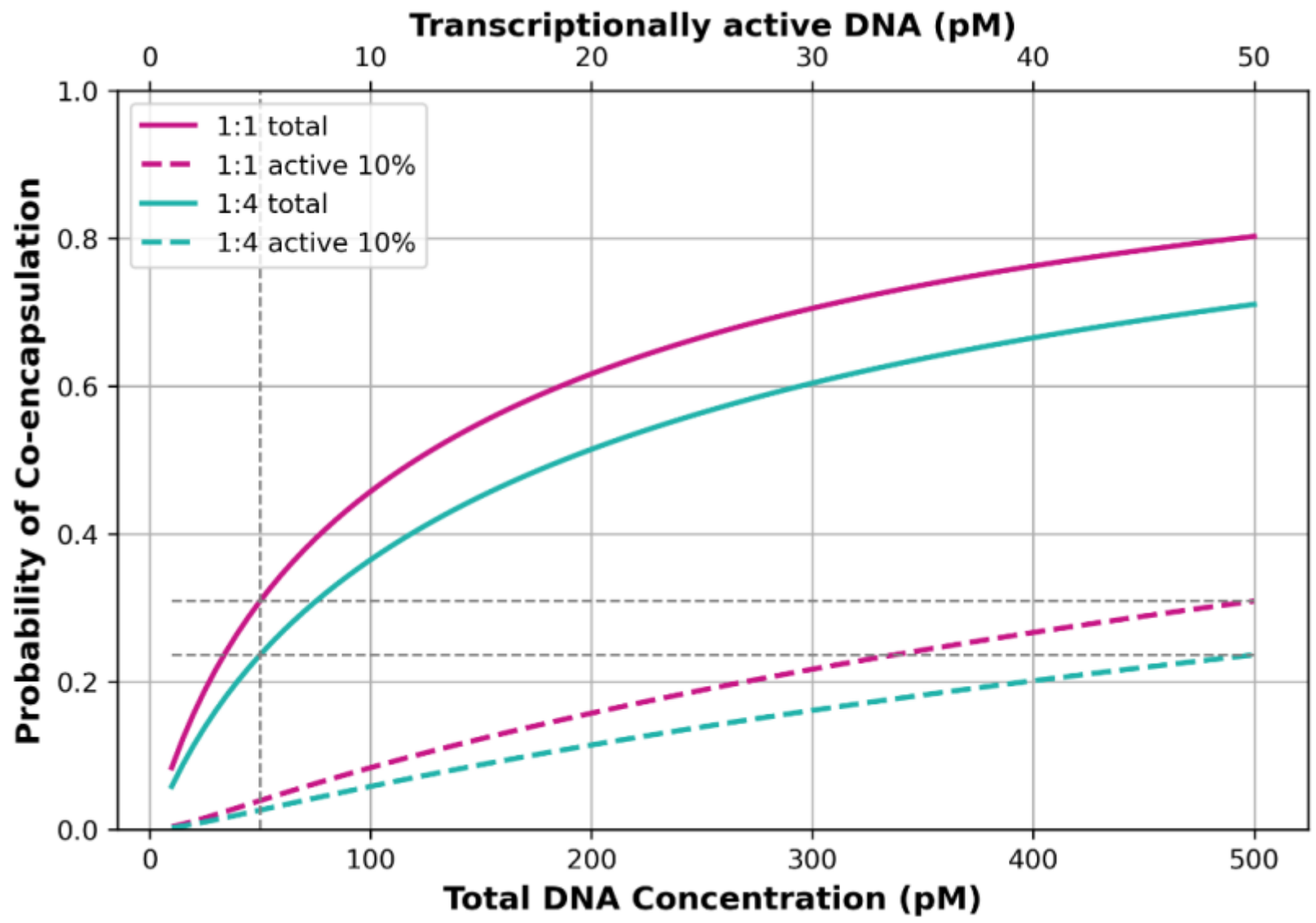

Modified Supplementary Fig. 6

We made the calculator available on GitHub so that users can enter the initial DNA concentrations of choice for A and B.
The code computes the average number of DNA copies per liposome, the probability of occupancy and the probability of co-encapsulation under different concentrations with two DNA species. The calculations are based on the empirical liposome size distribution according to the theoretical framework described in the Methods section. Nonetheless, the user can modify the compartment size parameters at will for a more general applicability. This code is publicly available on GitHub at https://github.com/DanelonLab/ACS.

A snapshot of the code for the 1:4 DNA ratio condition (corresponding to Fig. 5) is shown below (numerical values change every time the code is run because *d* (diameter) values are generated new):

```python
# DNA concentrations in pM
C_A_pM = 100 # TO BE DEFINED BY THE USER
C_B_pM = 400 # TO BE DEFINED BY THE USER

# pM to M
C_A_M = C_A_pM * 1e-12
C_B_M = C_B_pM * 1e-12

# Compartment diameter distribution
mean_d = 4.5e-6
std_d = 2.3e-6

# Log-normal parameters
mu = np.log((mean_d**2) / np.sqrt(std_d**2 + mean_d**2))
sigma = np.sqrt(np.log(1 + (std_d**2 / mean_d**2)))

# Generate d values
d_values = np.random.lognormal(mu, sigma, 1000)

# Compute lambda for each species
lambda_A = (np.pi * NA * C_A_M * d_values**3 * conversion_factor) / 6
lambda_B = (np.pi * NA * C_B_M * d_values**3 * conversion_factor) / 6

print(f"Species A (100 pM) - Mean λ: {lambda_A.mean():.3f}, Std λ: {lambda_A.std():.3f}")
print(f"Species B (400 pM) - Mean λ: {lambda_B.mean():.3f}, Std λ: {lambda_B.std():.3f}")

# Probability of each species being present in a compartment (Poisson: P(k>=1))
prob_A = 1 - np.exp(-lambda_A)
prob_B = 1 - np.exp(-lambda_B)

# Probability that both species are co-encapsulated
prob_both = prob_A * prob_B
print(f"Mean probability that both species are in the same compartment: {prob_both.mean():.3f}")
```

```
Species A (100 pM) - Mean λ: 5.243, Std λ: 10.495
Species B (400 pM) - Mean λ: 20.971, Std λ: 41.980
Mean probability that both species are in the same compartment: 0.716
```

As suggested by the reviewer, we generalized the equation 6 in Methods and modified the text to better account for the different instances of co-encapsulation:

"For competition experiments involving two independent DNA species A and B, the probability that both species are present within a liposome is:

$$P_{\text{both}} = P_{\text{A}\geq 1} \times P_{\text{B}\geq 1} = \left(1 - e^{-\lambda_{\text{A}}}\right) \times \left(1 - e^{-\lambda_{\text{B}}}\right),$$

where λ_A and λ_B are the expected number of DNA molecules of species A and B per compartment. Figure S6 illustrates the probability of both DNA species coexisting within the same liposome as a function of DNA concentration for 1:1 (experiments in Fig. 3c,d and Fig. 6e) and 1:4 (experiments in Fig. 5) ratios. Additionally, Figure S6 illustrates the probability of co-encapsulation of active DNA molecules when only 10% of the total DNA population is transcriptionally active.

A code to compute the predicted λ values and (co-)encapsulation probabilities of two DNA species is available on GitHub at https://github.com/DanelonLab/ACS."

The modified Fig. S6 displays the probability of co-encapsulation of two DNA species in the same liposome for a 1:1 and 1:4 DNA ratio (see page 8 in this letter).

We observe a difference in the probability of co-encapsulation between a 1:1 ratio and a 1:4 ratio, which arises directly from the Poisson statistics of DNA encapsulation. In a 1:1 ratio, the DNA is evenly divided between the two species (e.g., 250 pM each at a total of 500 pM), resulting in similar λ values for both species and a high co-encapsulation probability (~0.815). In contrast, for a 1:4 ratio (e.g., 100 pM for species A and 400 pM for species B at the same total concentration), the lower-concentration species (A) has a smaller λ_A, reducing P_A. Even though species B is abundant, the probability that the scarce species A is also present limits the overall co-encapsulation probability (~0.727). Consequently, the less abundant species determines the likelihood that both species coexist in the same compartment.

Minor comments:

- Does the heat-inactivation of DNAse I compromise liposome integrity?
Reply: Heat-inactivation of DNase I may affect liposome integrity, but it does not compromise the encapsulated DNA which remains suitable for qPCR or sequencing-based quantification. In our previous study (Abil et al., 2024), we showed that the initial concentration of encapsulated DNA measured by qPCR agrees with the expected value based on the quantity and size of liposomes

- The "inactive" lacZ_E538Q mutant construct still shows some activity based on ref 19. A quick back-of-the-envelope calculation assuming typical sub microM expression levels of LacZ suggests that micromolar amounts of allolactose might produced during incubation. Those might be sufficient to inactivate LacI (Kd = 600 nM) and explain some of the background depression.
Reply: When lactose was supplemented to the reaction (Fig. 4 and Fig. S8, Condition 3), expression was largely restored for the wild-type template, in line with our expectation that β-galactosidase could convert lactose into allolactose, derepressing gene expression. For the E538Q mutant, a slight derepression was observed (Fig. 4 and Fig. S8, Condition 3). The E538Q mutant has been reported to retain residual enzymatic activity (old ref. 19, now ref. 24) and may therefore contribute to partial derepression, as suggested by the reviewer. To acknowledge this possible mechanism, we added on page 10:
"For the E538Q mutant, a slight derepression was observed, which may arise from residual activity of the E538Q enzyme[24]."
Alternatively, this effect may arise from background conversion of lactose into allolactose in PURE system. Indeed, we routinely observe a slow (within 1 hour) change of color upon CPRG addition in control samples that do not contain DNA (Fig. S9). Nonetheless, this derepression was less pronounced than upon expression of the wild-type template.
While replication of the wild-type template was restored upon addition of lactose, the mutated template did not amplify (Fig. 4e, Condition 3). The difference of amplification fold between the

Conditions 2 (Repression) and 3 (Derepression) is not significant with the mutant enzyme (see table below).

	rep 1	rep2	rep3	Two-tailed P value (paired *t* test)	Significance
MUT condition 2	1.433	1.224	1.248	0.2679996	n.s.
MUT condition 3	1.433	1.724	1.418		

These data are not displayed in Figure 4e.

This result confirms that β-galactosidase-mediated allolactose production is essential for replication and the slight derepression induced by the mutant enzyme does not compromise autocatalytic selection of the wild-type enzyme in competition assays, both with a mock library (Fig. 5) and with an NNS library of gene variants (new Fig. 5c,d).

- In the abstract and main text, the authors use the wording "synthesis" of a deoxynucleoside triphosphate. This wording can be interpreted as de-novo nucleotide biosynthesis. Since the GOI enables Gmk-mediated conversion of dGMP to dGTP (with NDP kinase providing the second phosphorylation) in the PURE system, this is more precisely in situ activation/regeneration via salvage triphosphorylation rather than de-novo synthesis. I suggested to edit the abstract:, e.g. "ACS of three functions: transcription, in situ triphosphorylation that regenerates dGTP from dGMP to support DNA replication, and β-galactosidase activity." for reasons of clarity.

Reply: We agree and modified the abstract as: "we demonstrate ACS of three functions: transcription, ~~synthesis of a deoxynucleoside triphosphate~~ in situ regeneration of dGTP from dGMP to support DNA replication, and β-galactosidase activity."

Moreover, we also modified another sentence, where the wording "synthesis" was also used: "By leveraging the metabolic coupling between Gmk-mediated dGMP conversion and replication, we have demonstrated that DNA replicators can be engineered to operate when a self-encoded enzyme enables the ~~synthesis~~ regeneration of the DNA building blocks, broadening the scope and modularity of our ACS platform."

Reviewer #2 (Remarks to the Author):

This manuscript presents a proof-of-concept (PoC) study in which the authors attempt to apply an integrated DNA replication–transcription–translation system (IVTTR), based on their previously developed phi29 DNA replication machinery combined with the cell-free PURE system, to the evolution of genes using a liposome-based platform. As genes of interest (GOIs) for validation, they first examined the T7 RNA polymerase and demonstrated that autocatalytic selection is observed when the concentration of T7 RNA polymerase in the PURE system is extremely low. Next, they selected beta-galactosidase as a GOI and found that selection occurs when LacI and its regulatory factor, lactose, are introduced to link the DNA replication system to expression. Finally, they targeted Gmk, which participates in the pathway converting dGMP to dGTP, and attempted a similar experiment. They found that substrate exchange between liposomes posed a bottleneck, but reported that the introduction of cholesterol into the liposome membrane partially suppressed this issue.

Similar studies include the group's own previous report in 2024 (Ref. 5), as well as the work of Okauchi and Ichihashi (Continuous cell-free replication and evolution of artificial genomic DNA in a compartmentalized gene expression system, ACS Synth Biol, 2021). Both studies reported continuous evolution of DNA polymerases using similar liposome-based systems. The latter paper has not been cited in this manuscript; it should be cited, and the Introduction should be revised accordingly. At present, the positioning of this work within and beyond the field is not clearly articulated.
Reply: We agree that the present work needs better positioning within the field. We substantially revised the introduction and made a comparative study of related RNA- and DNA-based mechanisms for replication or/and evolution (Supplementary Table 1). Note that continuous evolution of DNA polymerases, as reported by Okauchi and Ichihashi, was performed in droplet microcompartments, not in liposomes. This study should have been cited, and we revised the first paragraph of the introduction accordingly:
"More recently, two self-replicating DNA systems capable of Darwinian evolution have been created[3]; one through rolling circle amplification followed by homologous recombination[4], and the other through protein-primed replication of a linear DNA encoding the ϕ29 bacteriophage DNA polymerase (DNAP) and terminal protein (TP)[5]. Both systems could serve as a genetic platform for integrating new functionalities toward the development of an autonomous synthetic cell."

Additionally, we modified the title and replaced the generic term "in vitro" by "synthetic cells" to better position our study in the synthetic cell research field.

Unlike those earlier studies, which actually tested the evolution of proteins, the present work only presents PoC experiments for GOIs that are not directly involved in replication but only indirectly connected. Therefore, in terms of novelty and significance, this manuscript falls somewhat short. If continuous evolution exploiting the high mutation rate of replication enzymes,

or at least selection based on random mutagenesis, were demonstrated as in the previous reports, the results would be more suitable for publication in this journal.

Reply: We want to emphasize that – unlike earlier studies – the selectable gene *lacZ* and activity of its encoded enzyme β-galactosidase are NOT directly involved in replication nor in transcription and translation. This is precisely the most impactful and innovative aspect of our ACS scheme as potentially any GOI can be selected through the implementation of a biomolecular circuit coupled to the central dogma.

We fully engaged with the reviewer' suggestion and performed additional experiments demonstrating the selection of replicative variants from an NNS library of the *lacZ* E538 codon. Within only one round, we found that metabolically active variants were enriched. In the Results section, we added:

"Finally, to demonstrate selection from a pool of variants generated by random mutagenesis, we constructed an NNS-degenerate *lacZ* E538 codon library of the *ori-Op2Op3-lacZ* template via site-saturated mutagenesis, theoretically comprising 32 DNA variants covering all 20 amino acids plus one stop codon (Fig. 5c). Analysis of the initial library (t0 Fig. 5d, Fig. S12) revealed the presence of all 32 expected NNS codons, along with a small number of additional NNW variants present at low frequency, likely arising from primer synthesis or amplification errors. IVTTR reactions were conducted as detailed above, in the presence or absence of LacI and lactose. Under selection conditions, the GAG codon, encoding glutamate (E) at position 538, was enriched across three independent replicates (Fig. 5d, Fig. S12). Notably, GAG is not the codon present in the parental wild-type construct (GAA) but is a synonymous variant. The enrichment of GAG over GAA may be attributed to their different abundances in the initial library. Given the NNS design of the library, codons ending in A are expected to be absent or extremely underrepresented in the starting pool, which is consistent with the absence of GAA at t0. Overall, the results indicate that selection is based on metabolic activity, as the system amplifies variants that produce a functional β-galactosidase, independent of the specific codon used. As expected, under non-selective conditions lacking LacI and lactose, no enrichment was observed (Fig. S13)."

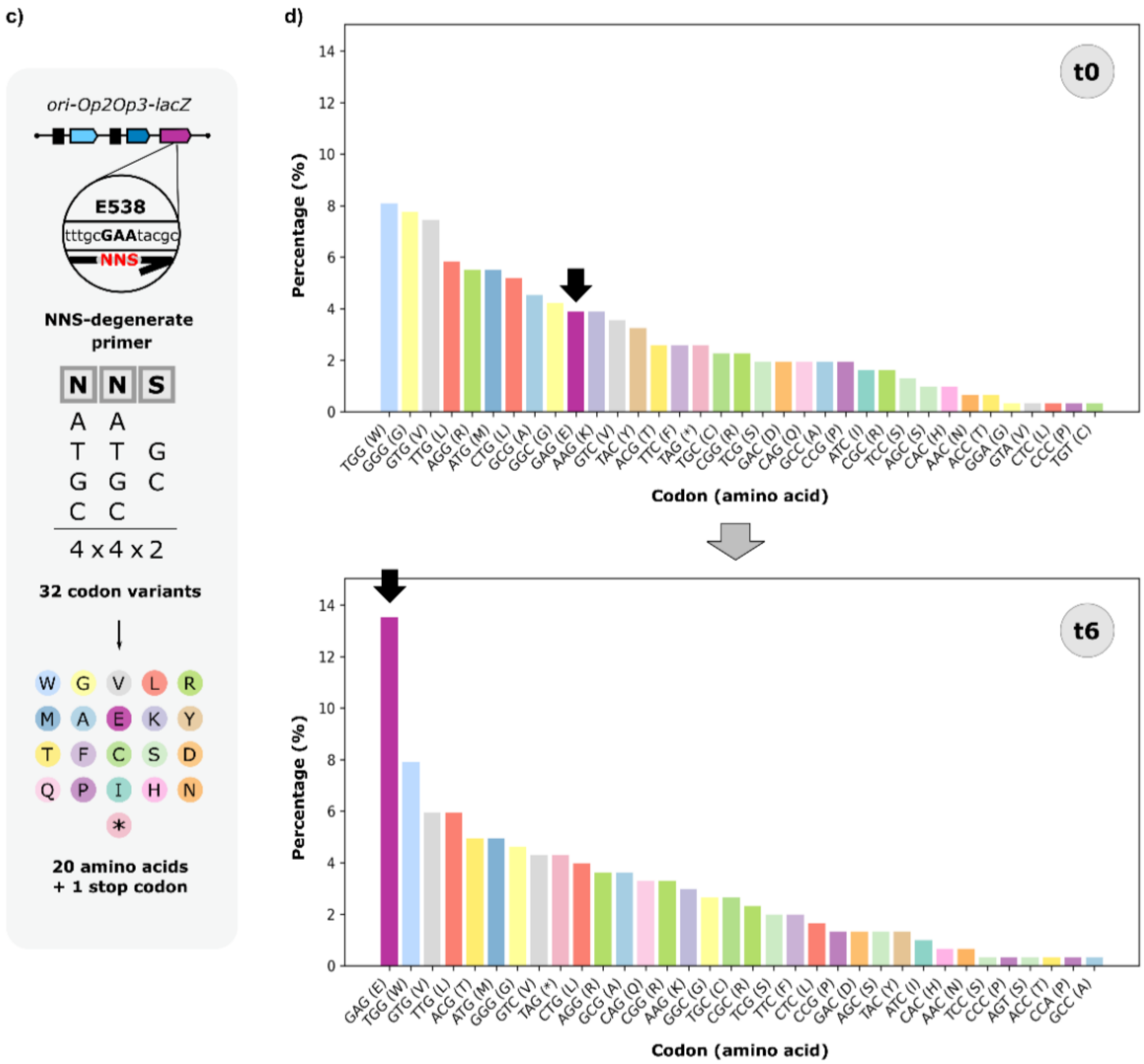

New Figure 5c,d

We emphasized this important result in the abstract: "In addition, we show that metabolically active replicators can be enriched from a library of variants generated by random mutagenesis."

Major Comment

- Data are presented suggesting that selection occurs even under conditions where multiple DNA molecules are present in a single liposome. As the authors themselves note in the Discussion, under the conditions of giant liposomes at 250 pM DNA concentration, co-encapsulation of two DNA species is expected. Nevertheless, as shown in Fig. 3c, the two DNA templates exhibit different amplification efficiencies, which may represent novel findings. However, in Fig. 5a, the figure appears to imply single-copy DNA encapsulation, making it unclear whether the experiments were actually performed at the single-copy level or not. This point needs to be clarified. If all experiments were performed at 250 pM DNA concentration, then the fact that DNA enrichment occurs under such conditions could indeed constitute a novel insight. Investigating the DNA concentration threshold at which selection occurs may provide an opportunity to present new and valuable findings.

Reply: First, to facilitate calculations of DNA copies per liposome and encapsulation probabilities under different bulk concentrations with one or two DNA species, we wrote a code that is publicly available on GitHub. One can compute the predicted co-encapsulation probabilities given the experimental liposome size distribution according to the theoretical framework described in the Methods section.
In Methods, we added: "A code to compute the predicted λ and (co-)encapsulation probabilities of two DNA species is available on GitHub at https://github.com/DanelonLab/ACS."

"Additionally, Figure S6 illustrates the probability of co-encapsulation of active DNA molecules when only 10% of the total DNA population is transcriptionally active."

In Fig. 5a, we drew single-copy DNA encapsulated in liposomes for simplicity. We understand this could be confounding given the many possible configurations of molecule encapsulation at these concentrations. Therefore, we clarified this point in the legends of Fig. 5a and Fig. S11: "The cartoon depicts only single-copy DNA encapsulated in liposomes at t0, but liposomes with no DNA or co-encapsulated DNA species are also expected."

At 250 pM of each DNA species (e.g., Fig. 3c), the expected probability that both DNA species coexist in the same liposome is:

$$P = \left(1 - e^{-\lambda}\right)^2 \approx 0.81$$

This is assuming that the two DNA species are 'independent' and that all DNA molecules are transcriptionally active. On that latter assumption, we wrote in the original manuscript: "*it is important to note that only a fraction of encapsulated DNA may be transcriptionally active*[27]*. While co-encapsulation may still happen, the probability that both active templates coexist within the same liposome is therefore lower than that calculated from the total DNA amounts, enabling ACS to proceed.*" In the modified Fig. S6, we also plotted the co-encapsulation probabilities vs total DNA assuming 10% active DNA.

In Fig. 3c, the fact that ori-tag-p2p3 and ori-T7-p2p3 differently replicate in favor of the longer T7RNAP-encoding template strongly suggests single-copy <u>active</u> DNA encapsulation; otherwise, the shorter ori-tag-p2p3 template would be preferably amplified.

To our knowledge, this is only the second report (after ref. 5) that not all DNA molecules (as measured by Nanodrop or Qubit) are active for gene expression. The implication may be important when designing directed evolution experiments in liposomes.
Alternatively, one could speculate that spatial proximity occurring when proteins are expressed from a DNA molecule favor interaction/reaction with that same DNA. For instance, a T7 RNAP produced from a given DNA would have a higher chance to replicate its own template. Or a β-galactosidase synthesized from a DNA molecule and producing allolactose would more efficiently derepress the expression of genes from the same template. This may contribute to segregating effects from different DNA molecules even when they are present in the same liposome. However, such effects are not visible in bulk reactions (e.g., the T7 RNAP

experiments) and, in the absence of stronger experimental data supporting this hypothesis in liposomes, we prefer to not discuss about this mechanism in the manuscript.

We performed experiments with 50 pM (instead of 500 pM in Fig. 3a) of ori-tag-p2p3 and ori-T7-p2p3 in separate liposome populations (figure below). At such a DNA concentration, the probability that two copies of DNA are co-encapsulated is lower (0.49 as calculated with the provided code), further reducing the co-encapsulation probability of two DNA species in competition assays.

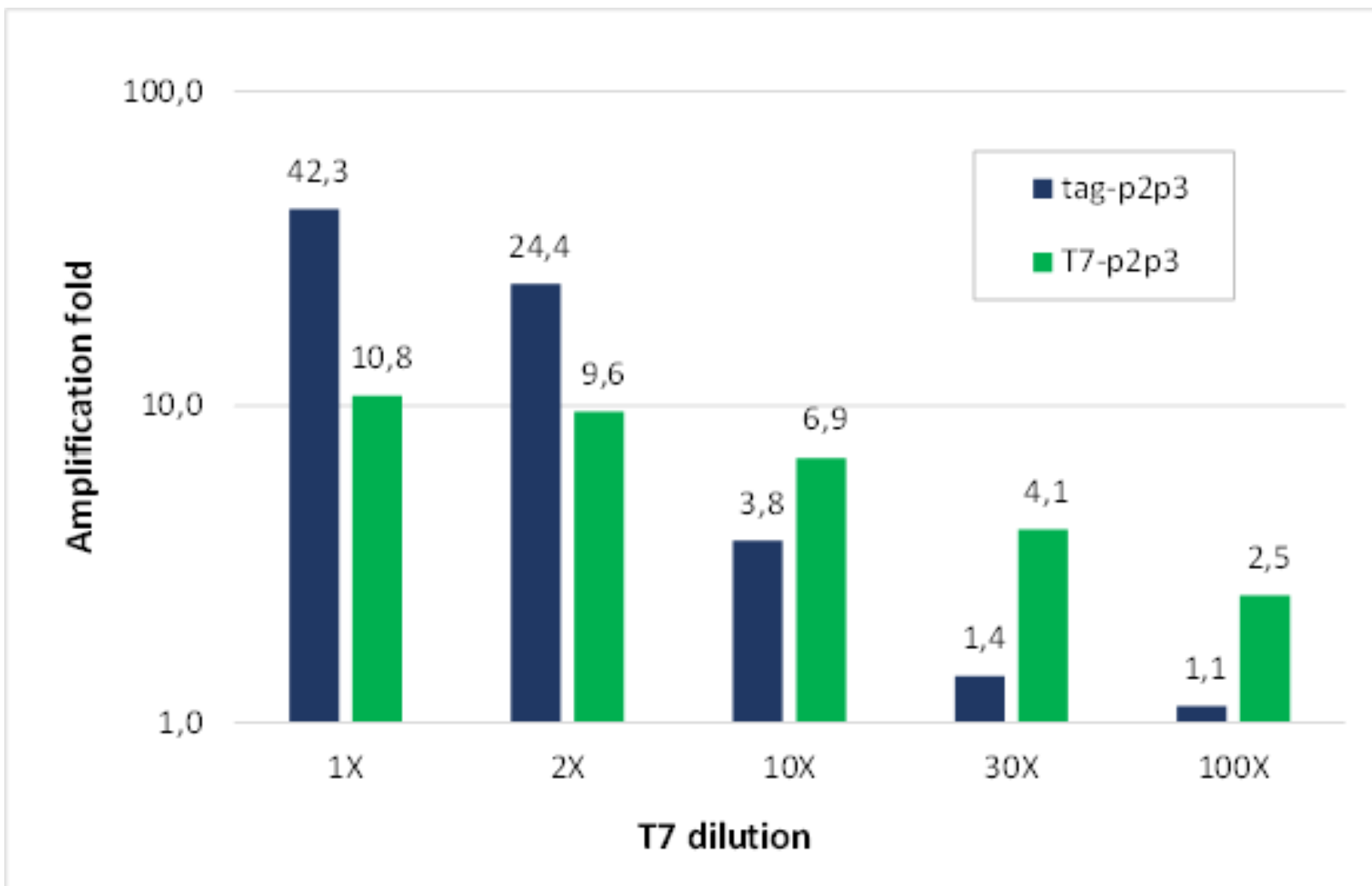

The trend of the amplification fold as a function of the concentration of purified T7 RNAP is similar as with 500 pM DNA (Fig. 3a): when purified T7 RNAP becomes limiting, replication is maintained only when T7 RNAP is regenerated from ori-T7-p2p3. Considering the tradeoffs for DNA concentration, selection pressure stringency and replication efficiency, we decided to use 500 pM total DNA and 30x diluted T7 RNAP for the competition assay with mixed DNA (Fig. 3b-d). Despite the higher probability of DNA co-encapsulation at 500 pM total DNA (250 pM each) than at 50 pM, we found a good compromise between expression and selective replication yielding enrichment of ori-T7-p2p3 over ori-tag-p2p3. The optimal DNA concentration and selection pressure conditions are case specific and should be determined for every system investigated.

Minor Comments

Abstract

- References should not be included in the abstract.

Reply: We removed the references from the abstract.

- The claim that beta-galactosidase is 'unrelated to the Central Dogma' is misleading. Since beta-galactosidase functions as part of the Lac operator system also used in this manuscript, it is in fact closely related to the Central Dogma.

Reply: We disagree; β-galactosidase is not a universal component for the flow of genetic information (central dogma). We designed a genetic circuit to couple the enzymatic activity to DNA replication. The evolvable function is the conversion of lactose into allolactose (it could virtually be any other reaction), while the introduction of the lac operator site links this function to the central dogma for selection.

Introduction

- The paragraph in lines 23–37 should objectively describe the positioning of this work within the field. Instead, it begins with detailed descriptions of the authors' prior studies, giving the impression of a narrow, incremental development. It would be more effective to start from a broader perspective and emphasize the importance and novelty of this work in the context of the field.

Reply: We substantially modified this paragraph. In particular, we deleted the sentence referring to our previous work where we combined DNA replication and lipid synthesis. Instead, we described relevant studies about RNA-based and DNA-based replicators in PURE system.

Results

- The order of genes differs between figures: p2–p3–GOI in Fig. 1 (conceptual scheme), GOI–p2–p3 in Fig. 2 (T7 RNAP experiment), p2–p3–GOI in Fig. 4 (lacZ experiment), and GOI–p2–p3 in Fig. 6 (Gmk experiment). If there is a rationale for these different arrangements, it should be described; otherwise, it should be explicitly stated that there is no particular reason.

Reply: The genetic organization may influence IVTTR efficiency and more generally transcription levels of multicistrons (called DNA context dependence), although there exists at present no design rules to optimize the order of genes, as far as we know. The choice for the arrangement of genes in our constructs mainly resulted from practical cloning considerations. We have now included a clarifying statement in the Results section ("Autocatalytic selection design"), point #1:

"The GOI can be positioned at different locations within the construct and in either orientation. In this work, the different gene arrangements resulted from cloning constraints rather than specific functional considerations."
