# Supplementary material for: Autocatalytic selection of gene functions in synthetic cells
Source: Commun Biol. 2026 May 27;9:1124. doi: 10.1038/s42003-026-10372-z (PMC13493933; doi:10.1038/s42003-026-10372-z)
Supplement: Supplementary file 2 [file 42003_2026_10372_MOESM2_ESM.pdf]

Supplementary information

for

Autocatalytic selection of gene functions in synthetic cells

Laura Sierra Heras[1] and Christophe Danelon[1*]

[1]Toulouse Biotechnology Institute (TBI), Université de Toulouse, 31077 Toulouse, France

[*]Corresponding author. Email: danelon@insa-toulouse.fr

Content:

Page 2 Table S1. Overview of studies on RNA- and DNA-based autocatalytic systems

Page 4 Table S2. Amplification folds of the DNA mixture for the β-galactosidase competition assay

Page 5 Table S3. List of primers used for PCR

Page 8 Table S4. List of primers used for qPCR

Page 9 Table S5. List of plasmids

Page 10 Figure S1. Optimization of PURE composition for IVTTR reactions

Page 11 Figure S2. Workflow for constructing linear DNA self-replicators with integrated genes using yeast as a cloning platform

Page 12 Figure S3. Biological replicates of protein expression from *ori-tag-p2p3* or *ori-T7-p2p3* DNA template

Page 12 Figure S4. Biological replicates of PURE reactions for assessing activity of gene-encoded T7 RNAP

Page 13 Figure S5. Probability distributions for different numbers of DNA molecules encapsulated within the same liposome at varying DNA concentrations

Page 13 Figure S6. Probability of co-encapsulation of two DNA species in the same liposome, accounting for active DNA fraction.

Page 14 Figure S7. Encapsulation and expression of the wild-type *lacZ* transcriptional unit in liposomes at increasing DNA concentrations

Page 15 Figure S8. Biological replicates of protein expression from *ori-Op2Op3-lacZ* (wild-type) or *ori-Op2Op3-lacZ_E538Q* (mutant) DNA template

Page 16 Figure S9. Background β-galactosidase activity in PURE system

Page 17 Figure S10. Assessing diffusion of allolactose between liposomes

Page 18 Figure S11. Autocatalytic selection of an active β-galactosidase encoded in the DNA self-replicator

Page 19 Figure S12. Biological replicates of selection from a *lacZ* E538 codon library in the presence of LacI and lactose.

Page 22 Figure S13. Biological replicates of selection from a *lacZ* E538 codon library in the absence of LacI and lactose.

Page 24 Figure S14. Biological replicates of protein expression from *ori-tag-p2p3* or *ori-gmk-p2p3* DNA template

Supplementary tables

Table S1. Overview of studies on RNA- and DNA-based autocatalytic systems

Ref.	Expression		Replication		Information storage		Coupling				Autocatalytic loop	Selection
	In vitro / in vivo	System	Yes / No	System	DNA / RNA	GOI and replication modules are on the same template (Yes / No)	Process 1: POI activity	Coupling molecule	Process 2: Expression	Process 3: Replication	Yes / No	Yes / No
6	In vitro	PURE system	Yes	Qβ RNAP	RNA	No	Nucleoside-diphosphate kinase: conversion of dNDPs to dNTPs	dNTPs	Translation of Qβ replicase and NDK*	Qβ- and NDK-coding RNA templates	Yes	Yes
10	In vitro	PURE system	Yes	Qβ RNAP	RNA	Yes	Nucleoside-diphosphate kinase: conversion of dNDPs to dNTPs	dNTPs	Translation of Qβ replicase and NDK*	Qβ-NDK-coding RNA template	Yes	Yes
11	In vitro	Cell lysate	No	None	DNA	No replication	Penicillin acylase (PAC): hydrolysis of phenylacetyl-methionine	Amino acid: Methionine	Translation of PAC	None	Yes	No (no coupling to DNA replication)
12	In vitro	PURE system	No	None	mRNA	No replication	Polylactate hydrolase (PLAse): hydrolysis of PLA into L-lactate	Amino acid precursor: L-lactate	Transalation of PLAase	None	Yes	No (no coupling to DNA replication)
13	In vitro	PURE system	No	None	DNA	No replication	Enzyme activities:<br>- Two CETCH cycle enzymes: involved in the conversion of CO2 into glyoxylate<br>- Glutamate-glyoxylate aminotransferase (GGAT): transamination of glyoxylate	Amino acid: Glycine	Transalation of:<br>- The two CETCH cycle enzymes<br>- GGAT	None	Yes	No (no coupling to DNA replication)
14	In vitro	PURE system	No	None	DNA	No replication	Aminoacylation and translation	-	Transalation of:<br>- 30 PURE translation factors<br>- GFP reporter	None	Yes	No (no coupling to DNA replication)
15	In vivo	*E. coli*	Yes	taq DNAP	DNA	No (purified taq DNAP)	Nucleoside-diphosphate kinase: conversion of dNDPs to dNTPs	dNTPs	Expression of NDK*	NDK-coding DNA template	Yes	Yes

* Expression is not linked to the activity of the protein of interest (POI) but it is required for autocatalysis.

Table S1. Overview of studies on RNA- and DNA-based autocatalytic systems (continued)

**Ref.**	**Expression**		**Replication**		**Information storage**		**Coupling**				**Autocatalytic loop**	**Selection**
	**In vitro / in vivo**	**System**	**Yes / No**	**System**	**DNA / RNA**	**GOI and replication modules are on the same template (Yes / No)**	**Process 1: POI activity**	**Coupling molecule**	**Process 2: Expression**	**Process 3: Replication**	**Yes / No**	**Yes / No**
16	In vivo	*E. coli*	Yes	taq DNAP	DNA	No	T7 RNAP: Transcription	-	Transcription of the Taq DNA polymerase	T7 RNAP-coding DNA template	Yes	Yes
							tRNA and/or tRNA synthetase: suppress amber codons in taq DNA polymerase	-	Translation of the Taq DNA polymerase (supression of amber codons)	tRNA/tRNA synthetase-coding DNA template		
17	In vivo	*E. coli*	Yes	taq DNAP	DNA	No (purified taq DNAP)	Nickase: DNA cleavage to produce primers	DNA primers	Expression of nickase*	Nickase-coding DNA template	Yes	Yes
18	In vitro	PURE system	Yes	Φ29 DNAP	DNA + mRNA	No	tRNA: amino acid donor	-	Translation of Φ29 DNAP	tRNA-coding DNA template	Yes	Potentially (not implemented in the study)
19	In vitro	PURE system	Yes	Φ29 DNAP	DNA	No	Aminoacyl-tRNA Synthetases (aaRSs): linking amino acids to their cognate tRNAs	-	Translation of Φ29 DNAP	aaRS-coding DNA templates	Yes	Yes
20	In vitro	PURE system	Yes	Φ29 DNA replication machinery (protein primed)	DNA	No	DNK: Conversion of dNMPs to dNDPs	dNTPs precursors: dNDPs	Expression of DNK, *p2* and *p3* genes*	DNK- and p2-p3-coding DNA templates	Yes	Yes
This work	In vitro	PURE system	Yes	Φ29 DNA replication machinery (protein primed)	DNA	Yes	T7 RNAP: Transcription		Transcription of T7RNAP, *p2* and *p3* genes	T7-p2p3-coding DNA template	Yes	Yes
							β-galactosidase: Conversion of lactose into alloctose	Derepressor: allolactose	Transcription of *lacZ*, *p2* and *p3* genes	lacZ-p2-p3-coding DNA template		
							GMK: Conversion of dGMP to dGDP	dNTP precursor: dGDP	Expression of GMK, *p2* and *p3* genes*	gmk-p2-p3-coding DNA template		

* Expression is not linked to the activity of the protein of interest (POI), but it is required for autocatalysis.

Table S2. Amplification folds of the DNA mixture for the β-galactosidase competition assay

Ratio (WT:MUT)	+ LacI + Lactose	Amplification folds measured by qPCR targeting the *p2* region	Amplification folds estimated from sequencing data
1:1	No	4.30	N/A
1:1	Yes	1.23	1.58
1:4	No	5.40	N/A
1:4	Yes	1.35	1.35

Table S3. List of primers used for PCR

Primer pair	DNA sequence (5'→3')	Purpose
ChD-1365	TTTGTTTAACTTTAAGAAGGAGATATACATATGAACACGATTAACATCGCTAAGAAC	Amplify the *T7 RNAP* gene from G442.
ChD-1366	AAAAAACTAACAGATACTCTAGTACAATTCTTACGCGAACGCGAAGTCC	
ChD-1363	GAATTGTACTAGAGTATCTGTTAGTTTTTTTCTACTAGAGTACTAGAG	Linearize G435 to clone the *T7 RNAP* gene or the *gmk* gene under PURE regulatory sequences.
ChD-1364	ATGTATATCTCCTTCTTAAAGTTAAACAAAATTATTTCTAGAGG	
ChDT-1	GAATTGTGAGCGGATAACAATTCCCCGGGAGACCACAACGGTTTCC	Linearize G435 with *lacO* overhangs upstream *p2*.
ChDT-2	TTGTTATCCGCTCACAATTCCCCTATAGTGAGTCGTATTAATTTCAC	
ChDT-19	CCCTCTGGAGACACCAGAGG	Linearize pT001 to introduce a *lacO* site upstream *p3*.
ChDT-20	CCCTATAGTGAGTCGTATTAGCAGTCG	
ChDT-21	TACGACTCACTATAGGGGAATTGTGAGCGGATAACAATTCCCCCCTCTGGAGACACCAG	Generate a dsDNA with a *lacO* site and overhangs to the linearized pT001.
ChDT-22	CTGGTGTCTCCAGAGGGGGGAATTGTTATCCGCTCACAATTCCCCTATAGTGAGTCGTA	
ChDT-7	TAGCATAACCCCTTGGGGC	Linearize G435 to clone *lacZ* under PURE regulatory sequences.
ChDT-8	TAAACAAAATTATTTCTAGACCCTATAGTGAGTCGTATTAATTTCAC	
ChDT-11	TTTGTTTTGGGTATGACCATGATTACGGATTCACTGGCCGTCGTTTTACAACGTCGTG	Amplify *lacZ* from pBADMyc-HIS-lacZ with start and stop codons.
ChDT-12	CGCCGTAGTCCCAATGAAAATTATTTTTGACACCAGACCAACTGG	
ChDT-9	GAAATAATTTTGTTTAACTTTAAGAAGGAGATATACATATGACCATGATTACGGATTCAC	Add overhangs to the *lacZ* gene amplicon for assembly into the G435 backbone.
ChDT-10	GGCCCCAAGGGGTTATGCTATTATTTTTGACACCAGACCAACTG	
ChDT-72	CCTTTGCCAATACGCCCACGCGATGG	Generate the *lacZ* E538Q (CAA > GAA) mutation in pT003.
ChDT-73	GCGTATTGGCAAAGGATCAGCG	
ChDT-199	TTTGTTTAACTTTAAGAAGGAGATATACATATGGCTCAAGGCACGCTTTATATTG	Amplify the *gmk* gene from *E. coli* genomic DNA.
ChDT-200	AAAAAACTAACAGATACTCTAGTACAATTCTCAGTCTGCCAACAATTTGCTG	

Table S3. List of primers used for PCR (continued)

ChDT-41	TTAGGGAGCACATCCATGCCAATAGCTCGACAAGCGGCGAGAG CCTTGCACCTATGCTATATAAAGTAAGCCCCCACCCTCAC	Amplify the *oriL-T7RNAP* and *oriL-gmk* fragments from the in vitro assembly products for in-yeast assembly of pY002 and pY011, respectively.
ChDT-42	TCAGCGTGTTGTAATGATGCGCCATGAATTAGAATGCGTGATGA TGTGCAAAGTGCCGTCGTGAGTCGTATTAGCAGTCGACG	
ChDT-43	GACGGCACTTTGCACATCATCACGCATTCTAATTCATGGCGCATC ATTACAACACGCTGATAATACGACTCACTATAGGGAGACC	Amplify the *p2p3-oriR* fragment from G435 for in-yeast assembly of pY002 and pY011.
ChDT-44	GCTACATCTTCCGTACTATGCTGTAGTCTCATGGTCGAGTTCTAT TGCTGTTCGGCGGCAAAAGTAGGGTACAGCGACAACATACAC	
ChDT-41	TTAGGGAGCACATCCATGCCAATAGCTCGACAAGCGGCGAGAG CCTTGCACCTATGCTATATAAAGTAAGCCCCCACCCTCAC	Amplify the *oriL-Op2Op3* fragment from pT002 for in-yeast assembly of pY001 and pY008.
ChDT-47	TCAGCGTGTTGTAATGATGCGCCATGAATTAGAATGCGTGATGA TGTGCAAAGTGCCGTCGGCAAAAAACCCCTCAAGACC	
ChDT-44	GCTACATCTTCCGTACTATGCTGTAGTCTCATGGTCGAGTTCTAT TGCTGTTCGGCGGCAAAAGTAGGGTACAGCGACAACATACAC	Amplify the *lacZ-oriR* fragment (from pT003) and the *lacZ_E538Q-oriR* fragment (from the in vitro assembly reaction) for in-yeast assembly of pY001 and pY008, respectively.
ChDT-48	GACGGCACTTTGCACATCATCACGCATTCTAATTCATGGCGCATC ATTACAACACGCTGACGCATGTGAAATTAATACGACTCAC	
ChDT-56	TGCCGCCGAACAGCAATAGAACTCGACCATGAGACTACAGCAT AGTACGGAAGATGTAGCCCTGGGTCCTTTTCATCACG	Amplify the *CEN6/ARS4-URA3* fragment from pRS316 for in-yeast assembly of pY001 and pY002.
ChDT-57	ATAGCATAGGTGCAAGGCTCTCGCCGCTTGTCGAGCTATTGGCA TGGATGTGCTCCCTAATCTGTGCGGTATTTCACACC	
ChDT-65	TGCCGCCGAACAGCAATAGAACTCGACCATGAGACTACAGCAT AGTACGGAAGATGTAGCGGTATCAGCTCACTCAAAG	Amplify the *ColE1-Amp-CEN6/ARS4-URA3* fragment from pRS316 for in-yeast assembly of pY008 and pY011.
ChDT-57	ATAGCATAGGTGCAAGGCTCTCGCCGCTTGTCGAGCTATTGGCA TGGATGTGCTCCCTAATCTGTGCGGTATTTCACACC	
ChDT-44	GCTACATCTTCCGTACTATGCTGTAGTCTCATGGTCGAGTTCTAT TGCTGTTCGGCGGCAAAAGTAGGGTACAGCGACAACATACAC	Introduce an NNS codon at residue E538 of *lacZ* via amplification from pT003.
ChDT-159	CCGCTGATCCTTTGCNNSTACGC	
ChDT-160	GCAAAGGATCAGCGGGCGCGTC	Linearize pY001 to incorporate the *lacZ* E538 NNS codon library.
ChDT-56	TGCCGCCGAACAGCAATAGAACTCGACCATGAGACTACAGCAT AGTACGGAAGATGTAGCCCTGGGTCCTTTTCATCACG	
ChDT-79	CGTCTGTGTCGCATGTGACCTTAGCATCCCTTCCCTTTGC	Amplify a 170-bp *tag* sequence from pRS316.
ChDT-80	CTATAGTGAGTCGTATTAATTGGTGTGGGTTTAGATGACAAGG	
ChDT-77	TCACATGCGACACAGACGAAGC	Linearize G435 to incorporate a *tag* sequence upstream *p2*.
ChDT-78	AATTAATACGACTCACTATAGGGAGACCAC	
ChDT-491E	5’-PHOS/AAAGTAAGCCCCCACCCTCACATGATACC	Generate 5’-phosphorylated linear templates for IVTTR.
ChDT-492E	5’-PHOS/AAAGTAGGGTACAGCGACAACATACACCATTTCC	

Table S3. List of primers used for PCR (continued)

ChDT-16	AAAGTAAGCCCCCACCCTCACATG	Amplify the *lacZ* transcriptional unit from pT003.
ChDT-17	AAAGTAGGGTACAGCGACAACATACAC	
ChD-980	ACGGCTGAAATTGACATCCCG	Amplify a region spanning part of *p3* and the *lacZ* transcriptional unit for Nanopore sequencing of diluted IVTTR samples.
ChD-1028	AGGTAATAAGACAACCAATCATAGGAGGCA	

Table S4. List of primers used for qPCR

Primer pair	DNA sequence (5'→3')	Purpose
ChD-976<br>ChD-977	GGATGAAGACTACCCGCTGC<br>ACAGGTCTGCGATTTCACCG	qPCR amplicon in *p2* gene
ChDT-81<br>ChDT-82	CCTTAGCATCCCTTCCCTTTGC<br>GGTGTGGGTTTAGATGACAAGG	qPCR amplicon in *tag*
ChDT-52<br>ChDT-53	CTGGAGAGATTCTTCGCAAGCG<br>GTAAGCGGAACTGACCGAGG	qPCR amplicon in *T7 RNAP* gene
ChDT-223<br>ChDT-224	GCAAATTCGCCAGAAGATGC<br>CTTGCGCCATACGCTTTGC	qPCR amplicon in *gmk* gene

Table S5. List of plasmids

Plasmid name	Relevant characteristics	GenBank accession number
G435	*ori-p2p3; AmpR; ColE1*<br>*p2* transcriptional unit: *T7p g10L RBS p2 vsv-r1 vsv-r2*<br>*p3* transcriptional unit: *T7p mod-g10L RBS p3 T7t*	
G442	*T7 RNAP* gene; *KanR; oriV*	
pRS316	*CEN6/ARS4-URA3; AmpR; ColE1*	
pBADMyc-HIS-lacZ	*lacZ* gene without start and stop codon; *AmpR; ColE1*	
pT001	*ori-Op2p3; AmpR; ColE1*<br>*p2* transcriptional unit: *T7p lacO g10L RBS p2 vsv-r1 vsv-r2*	
pT002	*ori-Op2Op3; AmpR; ColE1*<br>*p3* transcriptional unit: *T7p lacO mod-g10L RBS p3 T7t*	
pT003	*ori-lacZ; AmpR; ColE1*<br>*lacZ* transcriptional unit: *T7p g10L RBS lacZ T7t*	
pT007	*ori-tag-p2p3*; *AmpR; ColE1*	PZ358544
**In-yeast assembled plasmids**		
pY001	*ori-Op2Op3-lacZ*; *CEN6/ARS4-URA3*	PZ358545
pY002	*ori-T7-p2p3*; *CEN6/ARS4-URA3*<br>*T7 RNAP* transcriptional unit: *T7p g10L RBS T7 RNAP vsv-r1 vsv-r2*	PZ358543
pY008	*ori-Op2Op3-lacZ_E538Q*; *CEN6/ARS4-URA3; AmpR; ColE1*	PZ358546
pY011	*ori-gmk-p2p3*; *CEN6/ARS4-URA3; AmpR; ColE1*<br>*gmk* transcriptional unit: *T7p g10L RBS gmk vsv-r1 vsv-r2*	PZ358547

Supplementary Figures

a)

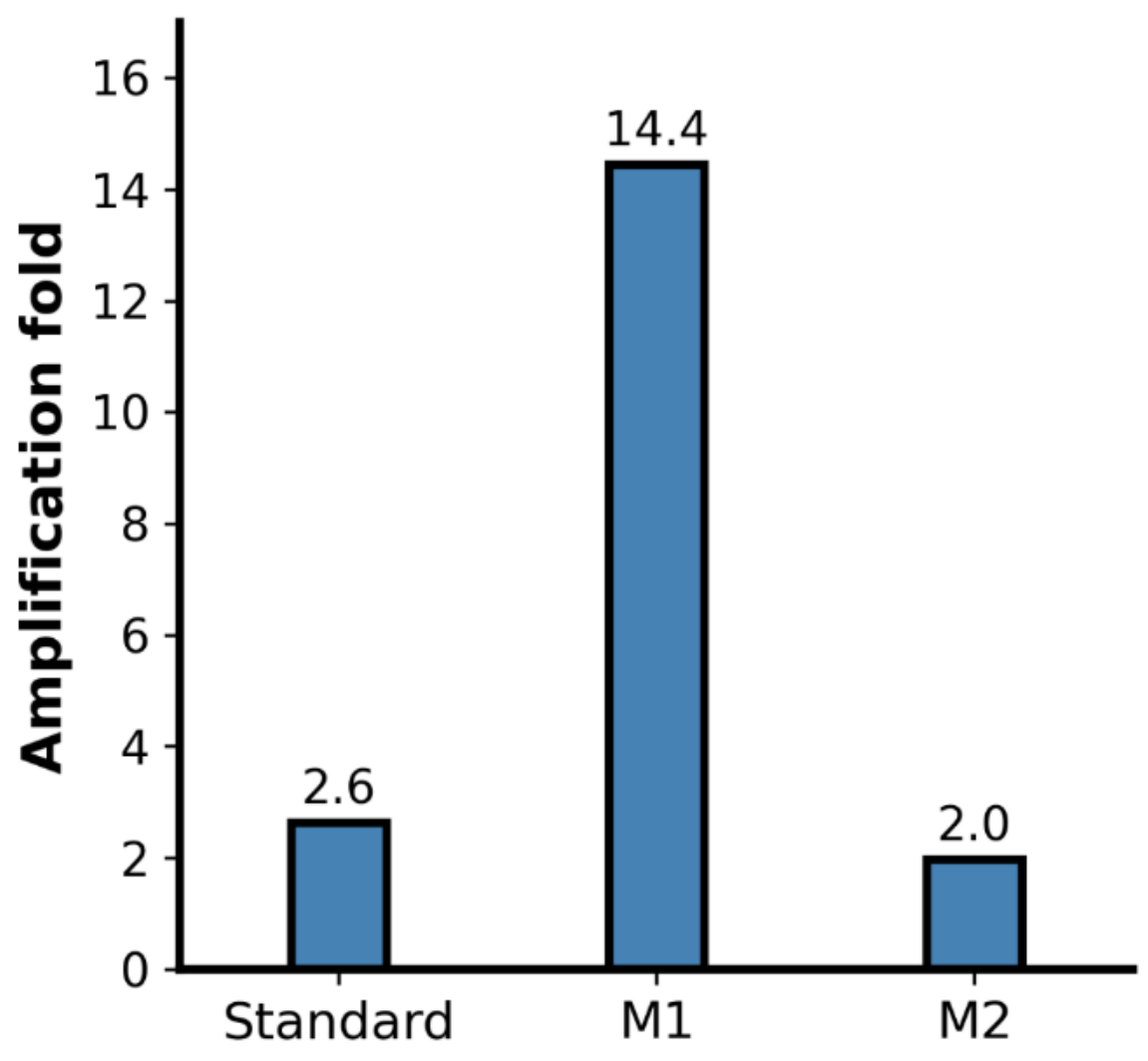

b)

Reagent name	M1 Factor Modified	M2 Factor Modified
HEPES	2.5	2.5
K-Glutamate	0.39	0.39
**Mg-acetate**	**0.58**	**1**
DTT	2	2
Spermidine	0.5	0.5
10-fTHF	1	1
CP	0.42	0.42
18 Aas	0.6	0.6
Tyr	1	1
Cys	1	1
**ATP/GTP**	**0.33**	**1**
**CTP/UTP**	**0.33**	**1**
tRNA mix	0.33	0.33

Fig. S1. Optimization of PURE composition for IVTTR reactions. **a**) Amplification folds from bulk IVTTR reactions using the standard PURE*frex*2.0 and two modified Solution I compositions. *Ori-p2p3* at 500 pM was used as template. **b**) Relative changes in concentration of the two modified compositions, M1 and M2, with respect to the standard PURE*frex*2.0. For example, a factor of 2 means that the concentration has been doubled, while a factor of 0.5 means the concentration has been decreased by half. Increases are shown in green and decreases in orange. The difference between M1 (active for IVTTR) and M2 (inactive for IVTTR) lies in the concentration of Mg-acetate and NTPs (bold), indicating these are critical factors for successful replication. Specific working concentrations are not disclosed for confidentiality reasons as agreed with GeneFrontier Corp. The optimized PURE*frex*-M1 is available from GeneFrontier as a customized kit. Supplementary Data is available for this figure.

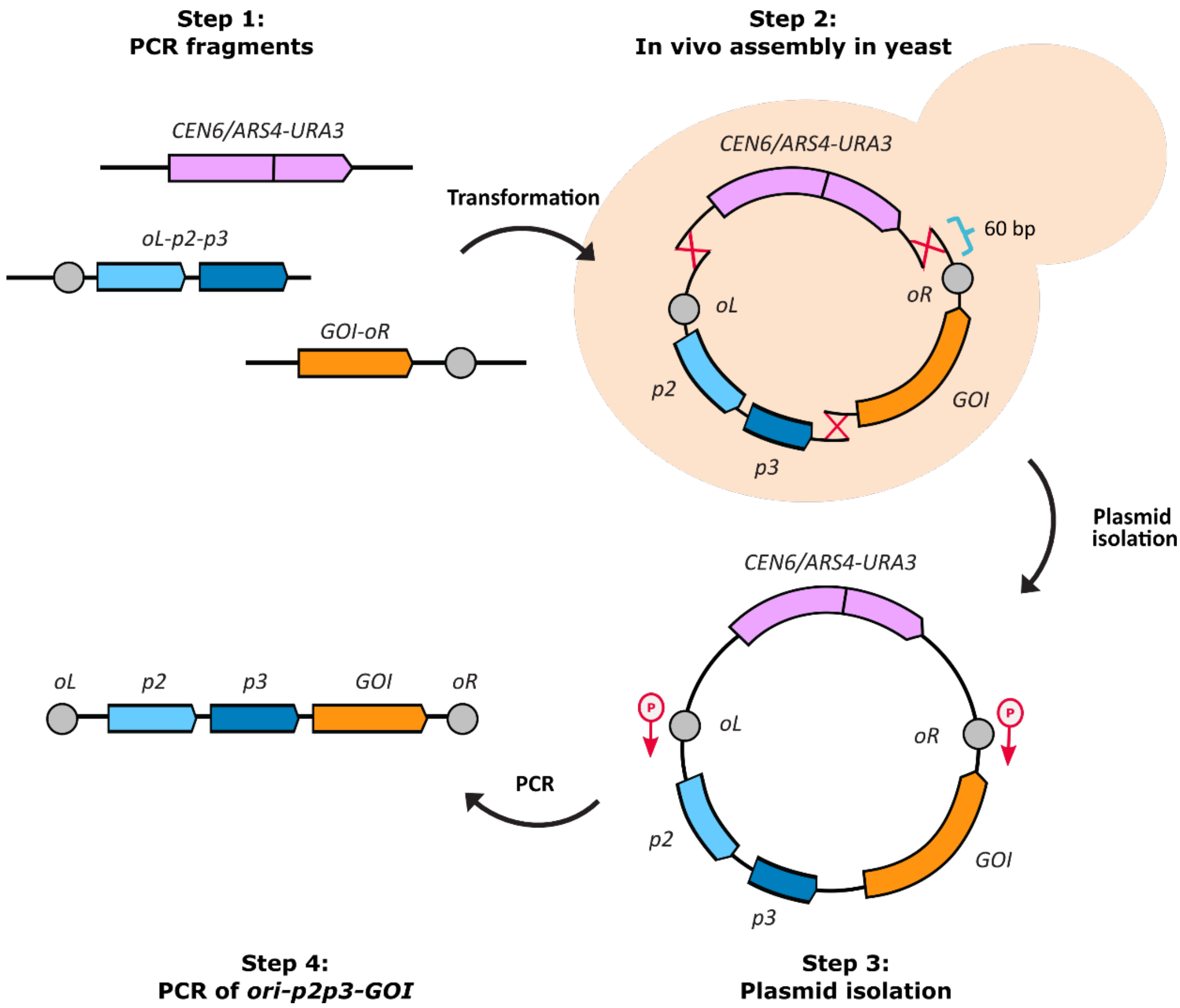

Fig. S2. Workflow for constructing linear DNA self-replicators with integrated genes using yeast as a cloning platform. The process consists of four steps: (1) Amplification of the genetic elements via PCR. This includes the *p2-p3* fragment and the gene of interest (GOI) fragment. Pre-cloning the GOI between ϕ29 replication origins ensures that one origin is already part of the fragment. The GOI can be introduced either upstream or downstream of the replication genes. A fragment containing a yeast origin of replication and selection marker (*CEN6/ARS4-URA)* is required for amplification and maintenance in yeast. (2) Transformation of yeast with the DNA fragments for in vivo assembly. The DNA fragments were designed with 60-bp synthetic overlaps for homologous recombination. (3) Isolation of the assembled plasmid. (4) Generation of linear self-replicators through PCR using 5′ phosphorylated primers.

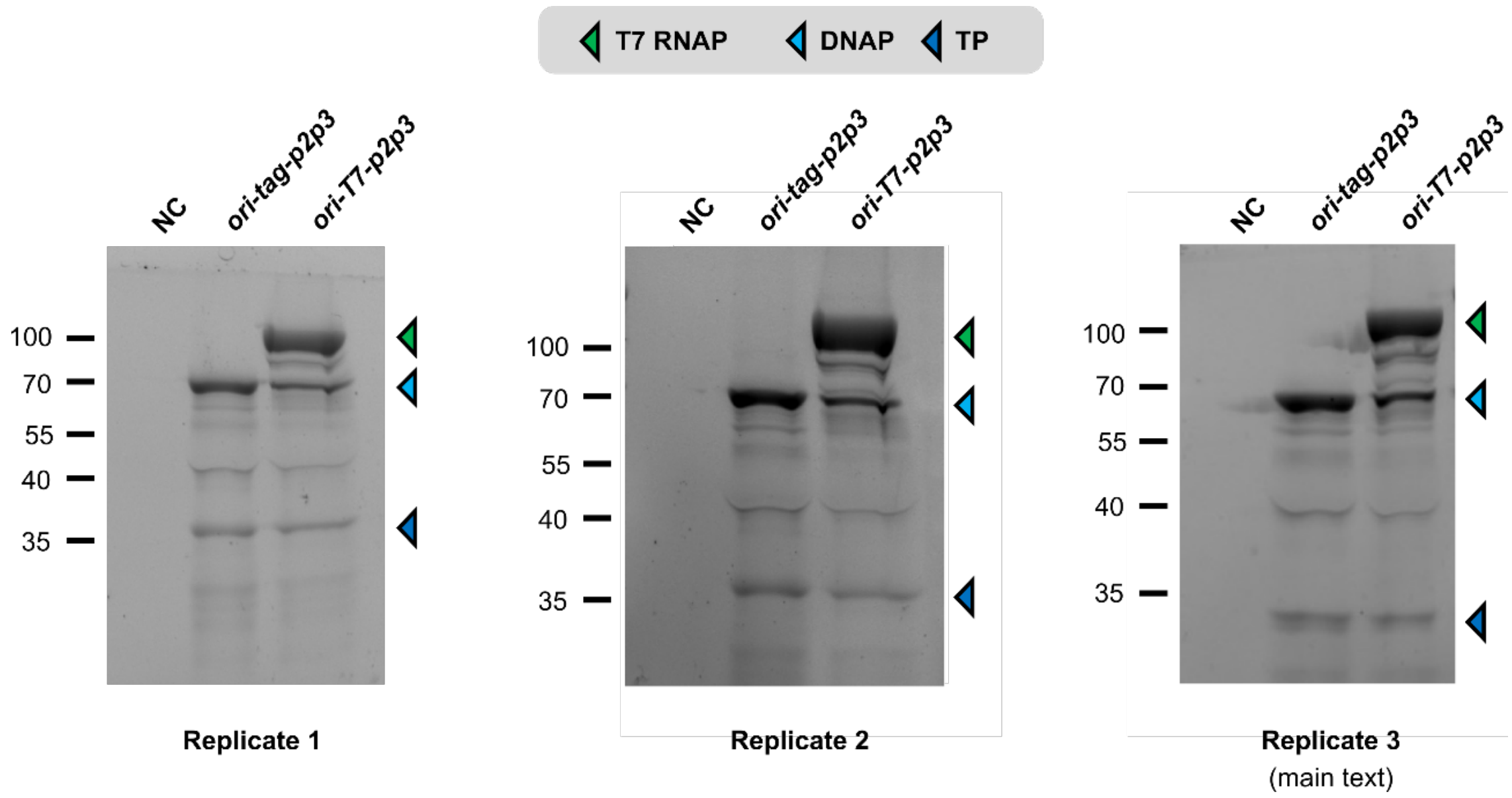

Fig. S3. Biological replicates of protein expression from *ori-tag-p2p3* or *ori-T7-p2p3* DNA template. PURE reactions were supplemented with GreenLys reagent and visualized on SDS-PAGE gels. T7 RNAP (~ 99 kDa), DNAP (~ 66 kDa), and TP (~ 31 kDa). NC: negative control with no DNA. Supplementary Data is available for this figure.

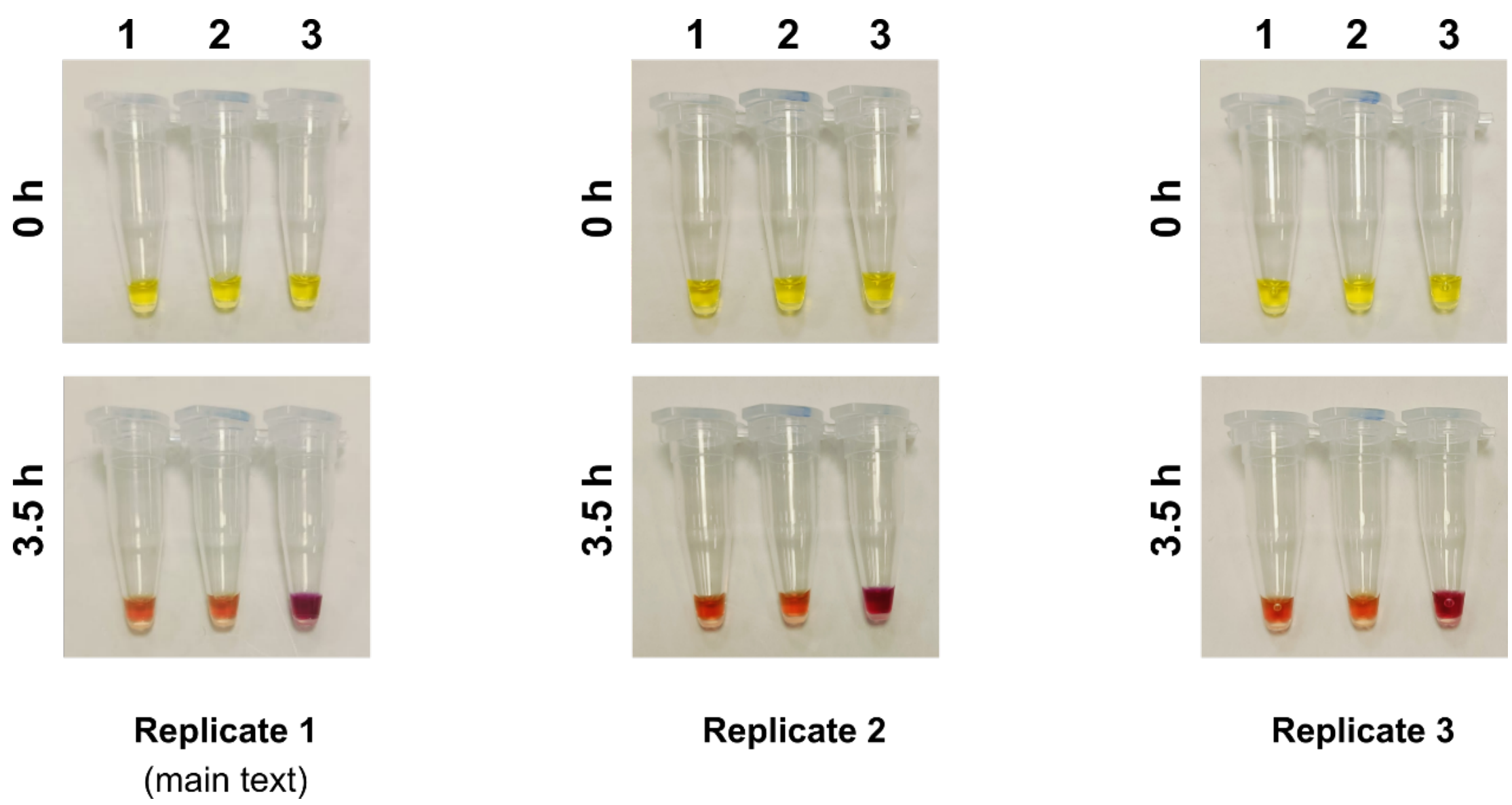

Fig. S4. Biological replicates of PURE reactions for assessing activity of gene-encoded T7 RNAP. The experiment was performed as described in Fig. 2c. Images were taken following CPRG addition (0 h) and 3.5 h later. (1) No DNA, (2) *ori-tag-p2p3*, and (3) *ori-T7-p2p3*. Supplementary Data is available for this figure.

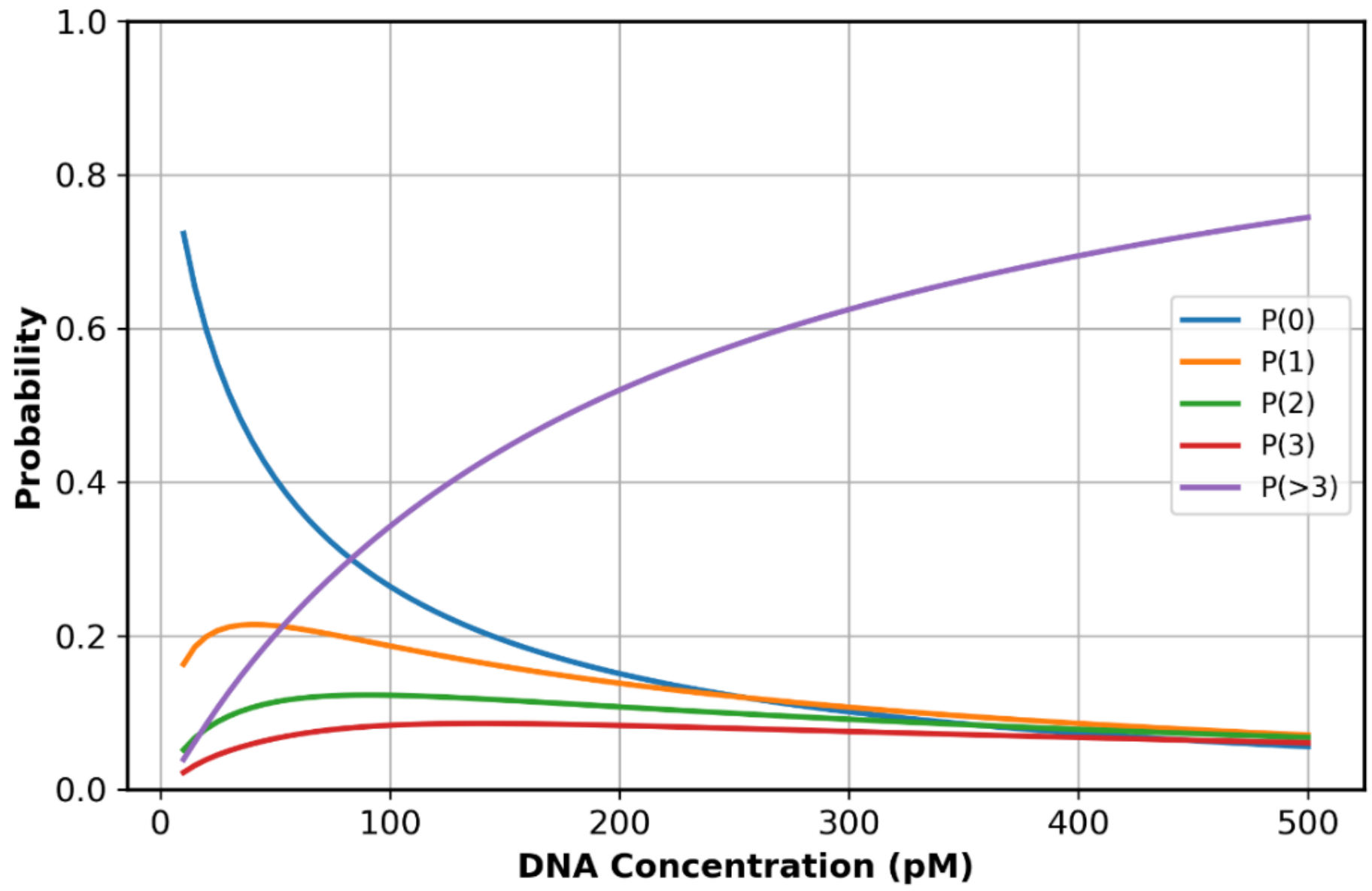

Fig. S5. Probability distributions for different numbers of DNA molecules encapsulated within the same liposome at varying DNA concentrations. The probabilities were calculated for 0, 1, 2, 3, and more than 3 molecules using a Poisson distribution. The liposome diameter is modeled using a log-normal distribution with a mean of μ_d = 4.5 µm and a standard deviation σ_d = 2.3 µm.

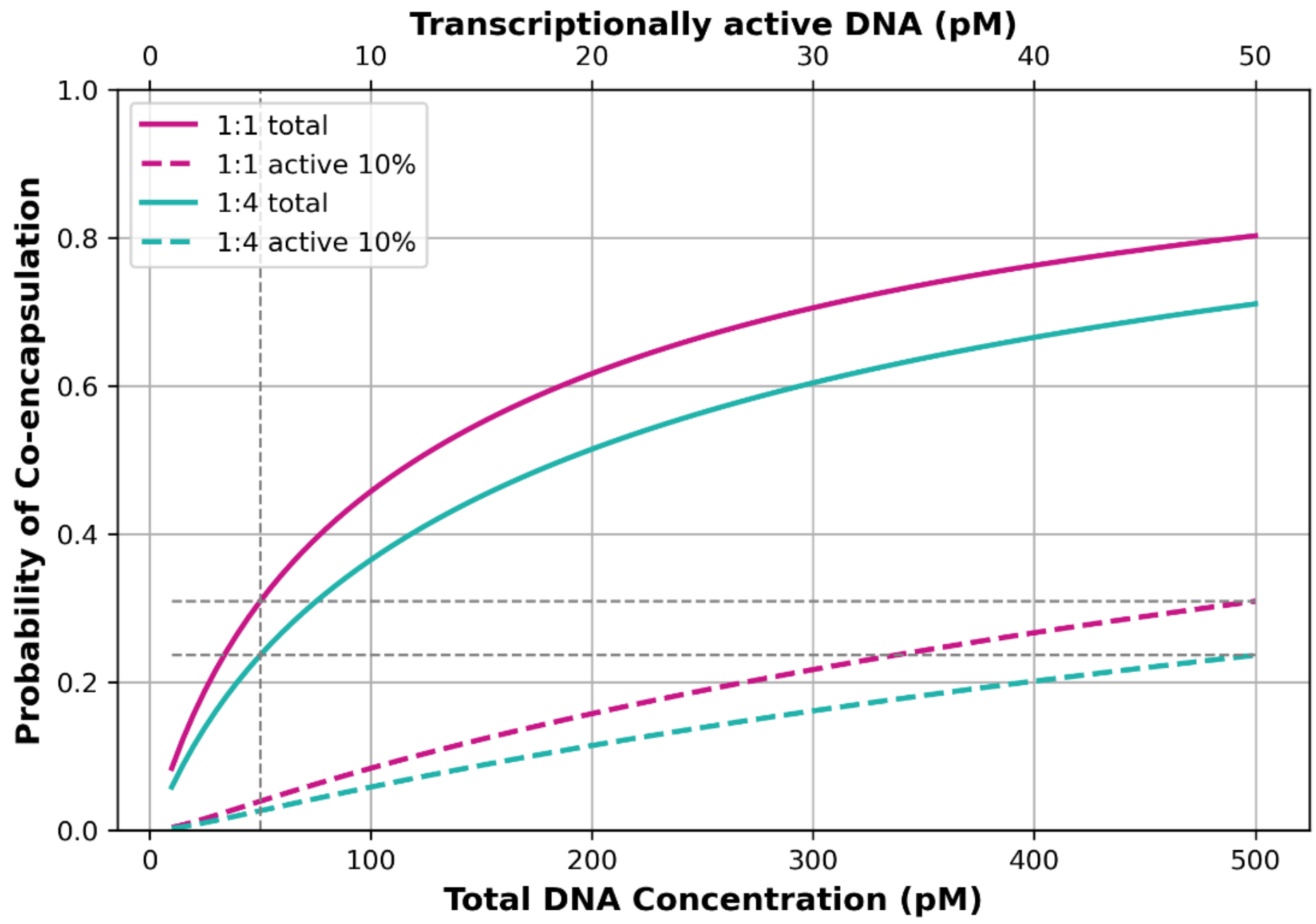

Fig. S6. Probability of co-encapsulation of two DNA species in the same liposome, accounting for active DNA fraction. The plot shows the probability that at least one copy of each DNA species is encapsulated within the same liposome, calculated for 1:1 and 1:4 DNA ratios. Solid lines represent the probability of co-encapsulation assuming all DNA molecules are active. Dashed lines represent the probability of co-encapsulation of active DNA molecules when only 10% of the total DNA population is transcriptionally active. The vertical dashed line marks a total DNA concentration of 50 pM; the corresponding probability matches that obtained at 50 pM active DNA (equivalent to 500 pM total DNA).

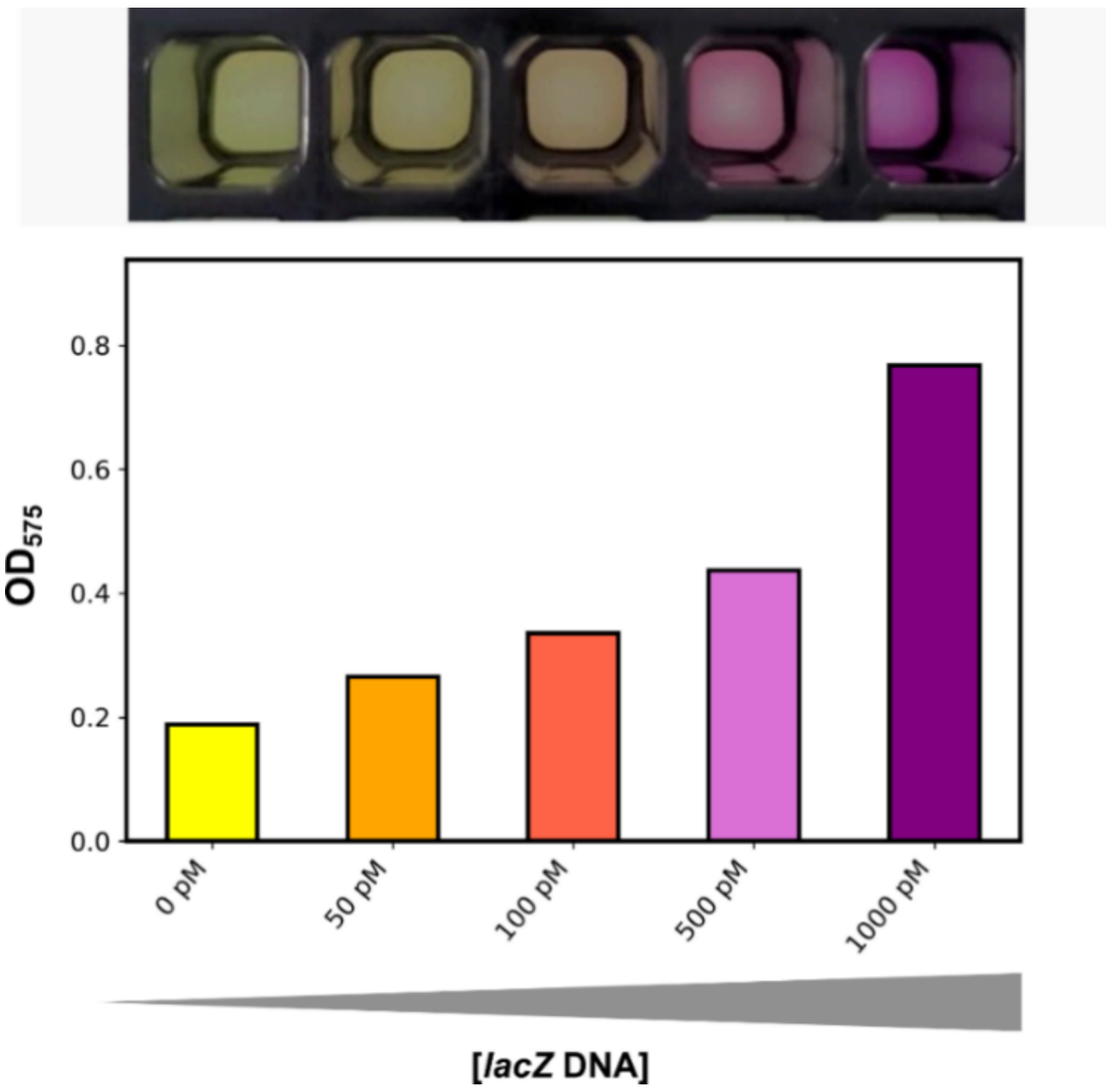

Fig. S7. Encapsulation and expression of the wild-type *lacZ* transcriptional unit in liposomes at increasing DNA concentrations. After liposome formation, CPRG was added to monitor β-galactosidase activity. Reactions were incubated for 2 h at 30 °C, after which images were taken (top) and OD_{575} values were measured (bottom). Both the color change and absorbance values correlate with the increasing amount of input DNA. Supplementary Data is available for this figure.

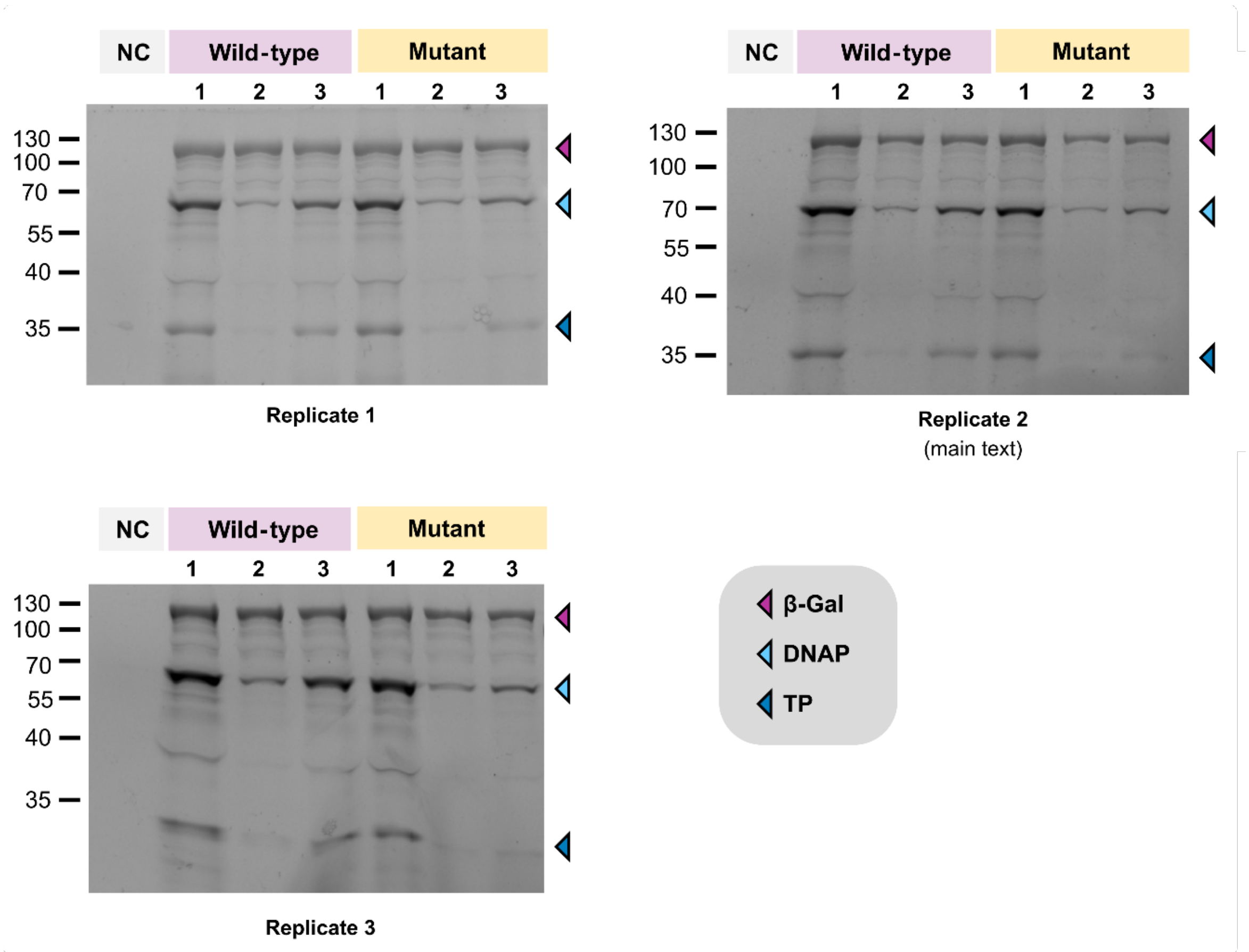

Fig. S8. Biological replicates of protein expression from *ori-Op2Op3-lacZ* (wild-type) or *ori-Op2Op3-lacZ_E538Q* (mutant) DNA template. PURE reactions were supplemented with GreenLys reagent and visualized on SDS-PAGE gels. Conditions: (1) in the absence of LacI; (2) in the presence of LacI; (3) in the presence of LacI and lactose. β-galactosidase (~ 117 kDa), DNAP (~ 66 kDa), and TP (~ 31 kDa). NC: negative control with no DNA. Supplementary Data is available for this figure.

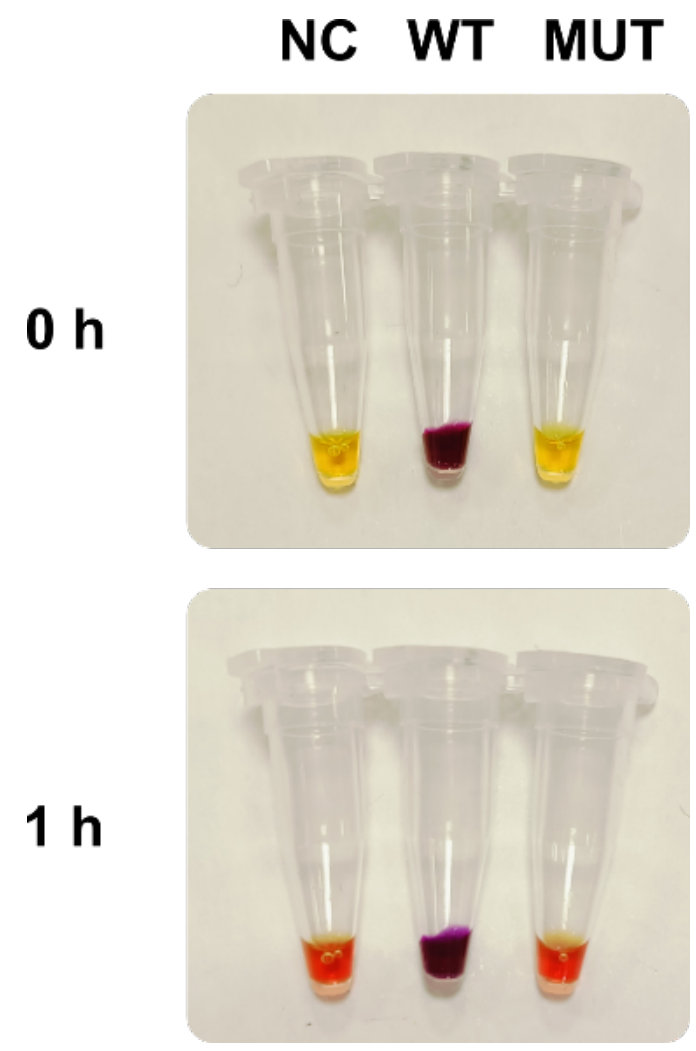

Fig. S9. Background β-galactosidase activity in PURE system. A progressive color change is observed in the negative control (NC) sample without a DNA template. The top image corresponds to Fig. 4b (main text) and was taken after 1 h of expression at 30 ˚C and immediately following CPRG addition. The bottom image was taken 1 h later, showing conversion in the NC sample, which indicates the presence of a β-galactosidase-like side reaction in PURE system. Notably, the NC and the mutated (MUT) template samples exhibit the same color change, suggesting that the observed effect is likely due to residual β-galactosidase activity in PURE rather than activity of the mutant enzyme. Supplementary Data is available for this figure.

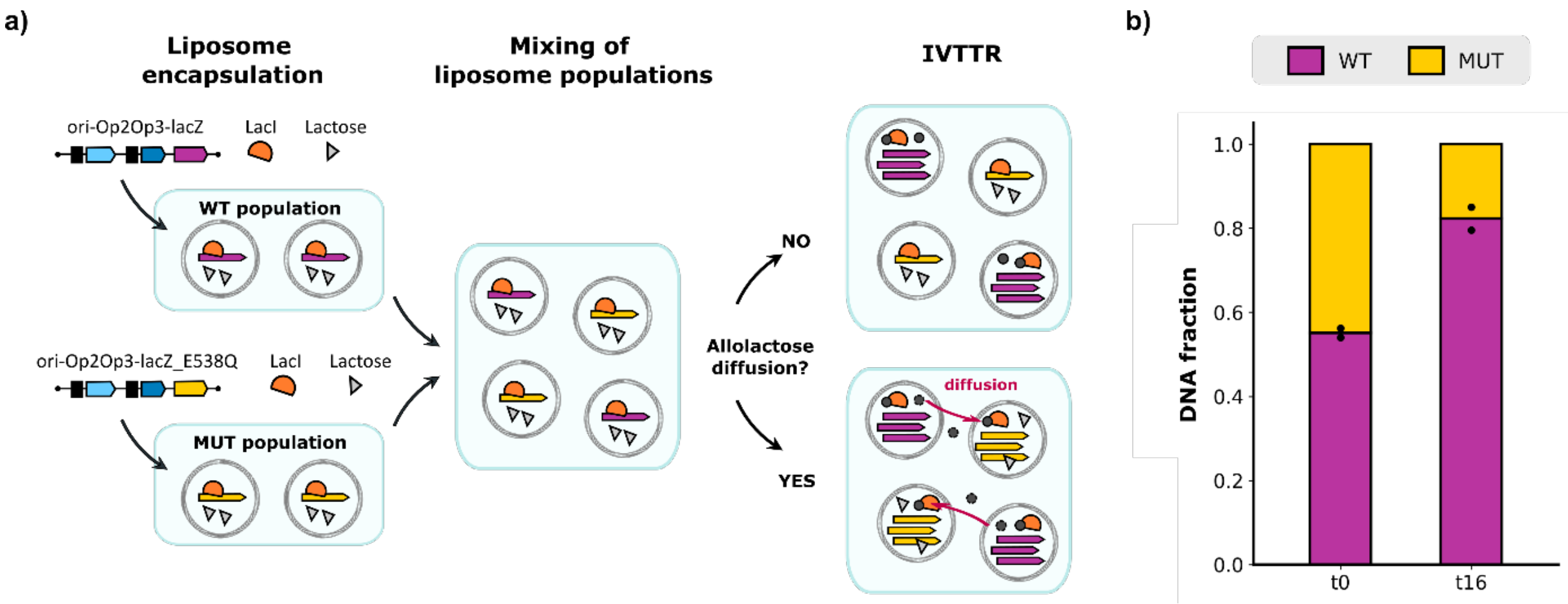

Fig. S10. Assessing diffusion of allolactose between liposomes. a) Schematic illustration of the experimental setup to evaluate allolactose diffusion. Two liposome populations were generated: one encapsulating the wild-type DNA template (*ori-Op2Op3-lacZ*) and the other containing the mutated DNA template (*ori-Op2Op3-lacZ_E538Q*), both in the presence of LacI and lactose. The populations were mixed in a 1:1 ratio and incubated for 16 h. In the absence of allolactose diffusion (top), only the wild-type template would replicate, maintaining the genotype-to-phenotype linkage. In contrast, if allolactose diffuses between liposomes, both templates would replicate, compromising the selection process. **b**) Fraction of wild-type (WT) and mutated (MUT) DNA templates in the population before (t0) and after (t16) IVTTR, as determined by long-read sequencing. Enrichment of WT over MUT after IVTTR indicates that allolactose diffusion across the liposome membrane is too low to compromise selection. Individual symbols represent data from two biological replicates (independent experiments). Supplementary Data is available for this figure.

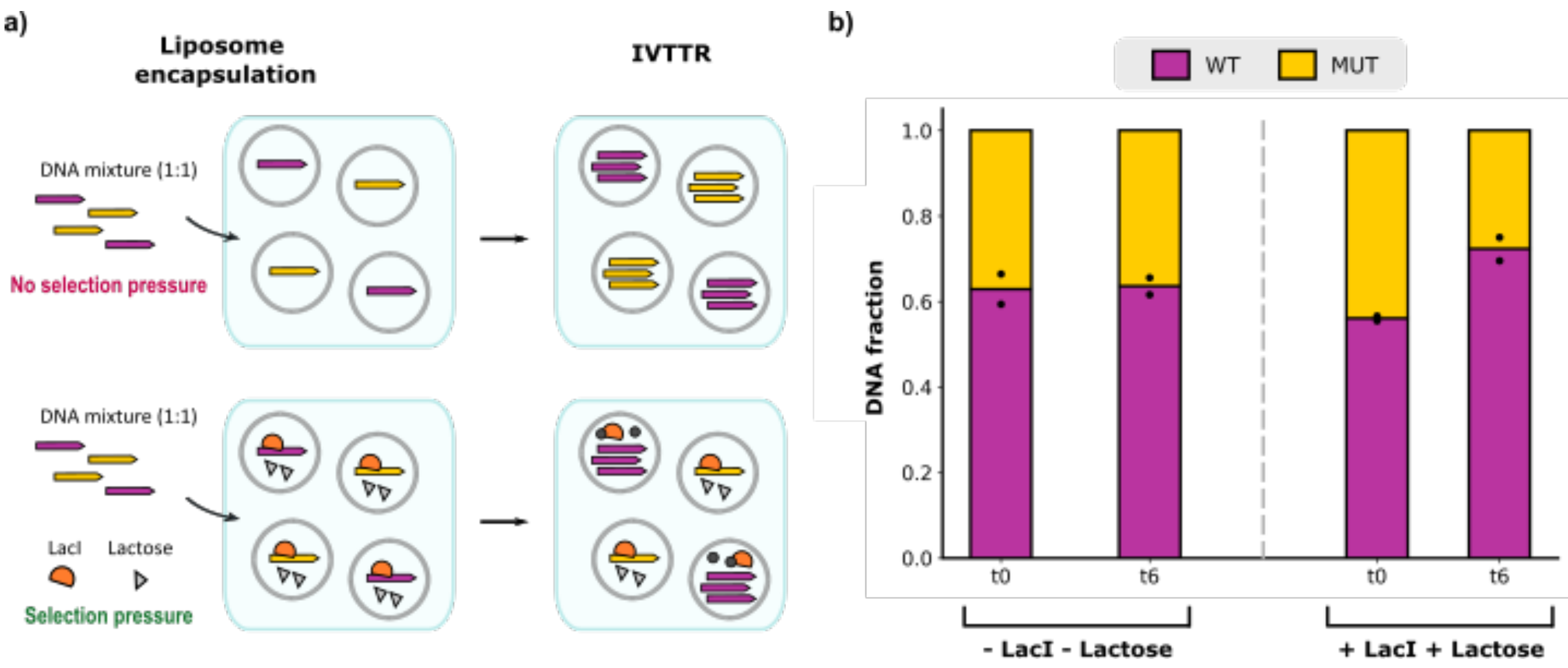

Fig. S11. Autocatalytic selection of an active β-galactosidase encoded in the DNA self-replicator. a) Schematic illustration of the experimental conditions and predicted replication outcomes. A 1:1 mixture of wild-type (purple) and mutated (yellow) DNA templates was encapsulated along with the IVTTR components into liposomes (total DNA concentration was 500 pM). In the absence of LacI (no selection pressure), both templates are expected to replicate. When LacI and lactose are co-encapsulated (selection pressure), only the wild-type template is expected to be de-repressed providing a replication advantage. The cartoon depicts only single-copy DNA encapsulated in liposome at t0, but liposomes with no DNA or co-encapsulated DNA species are also expected. **b**) Fraction of the wild-type (WT) and mutated (MUT) variants in the DNA mixture before (t0) and after (t6) IVTTR. The number of reads for each template, as determined by long-read Nanopore sequencing, was used to quantify the relative abundance of each variant. Individual symbols represent data from two biological replicates (independent experiments). Supplementary Data is available for this figure.

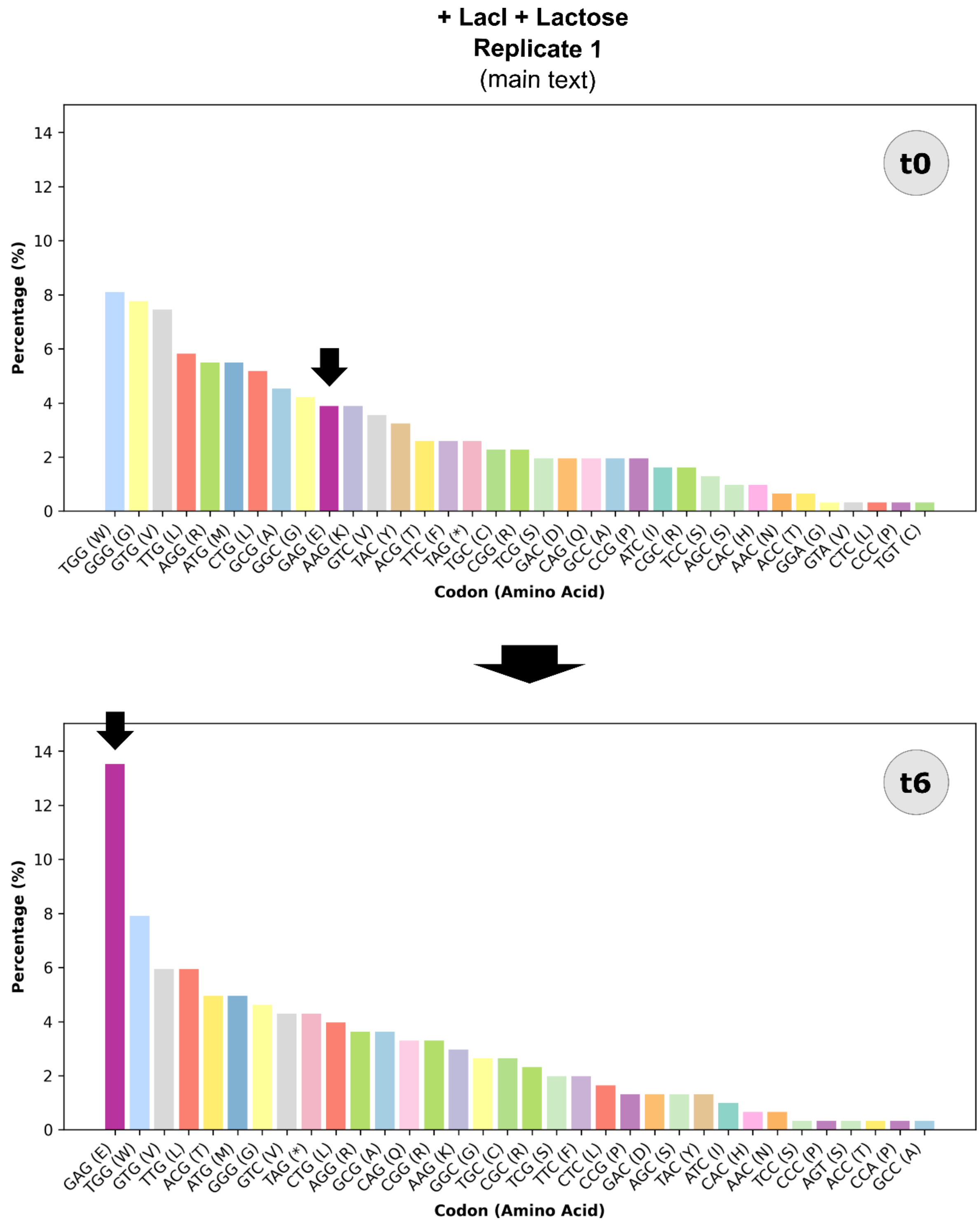

Fig. S12. *The figure continues on the next page.*

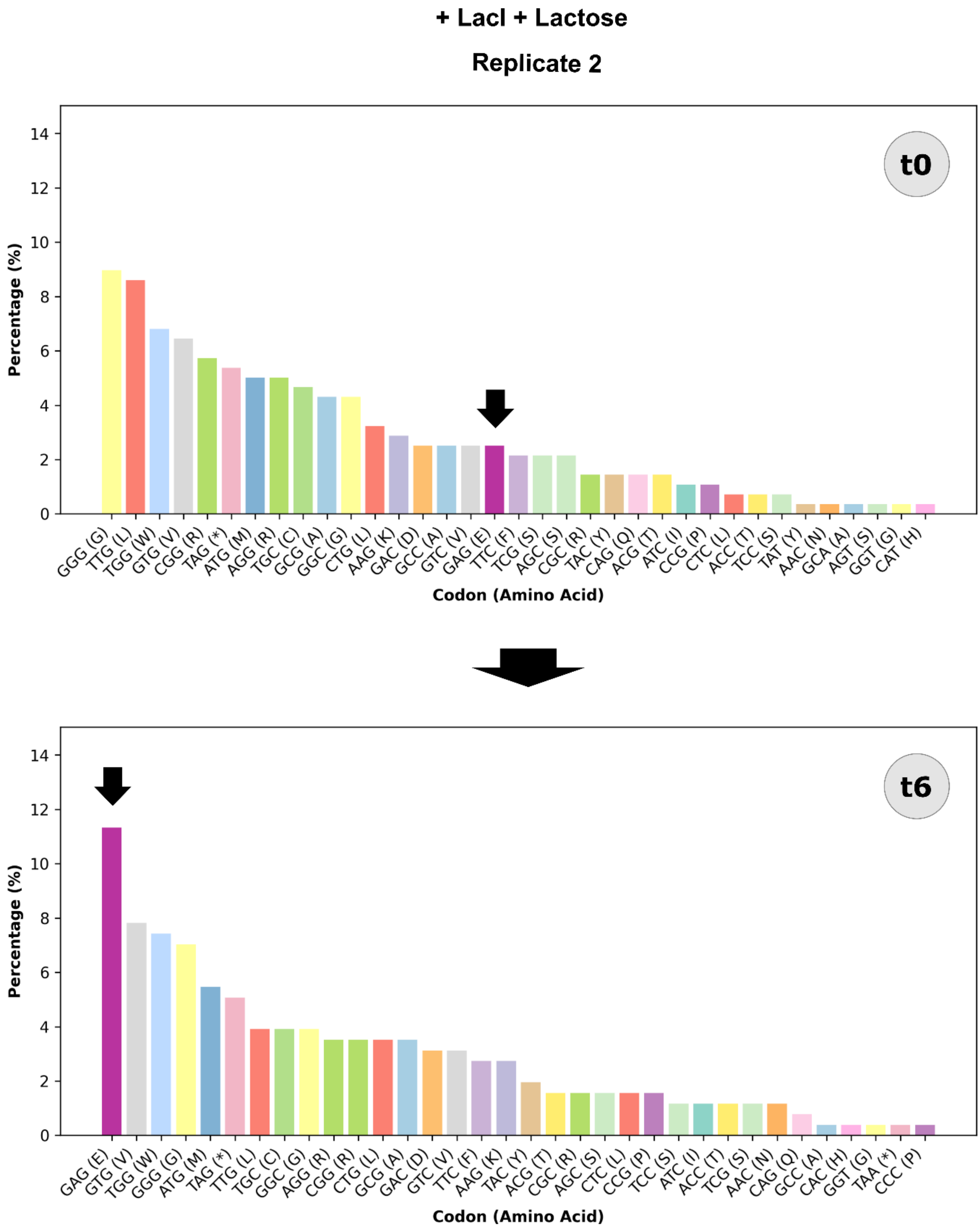

Fig. S12. *The figure continues on the next page.*

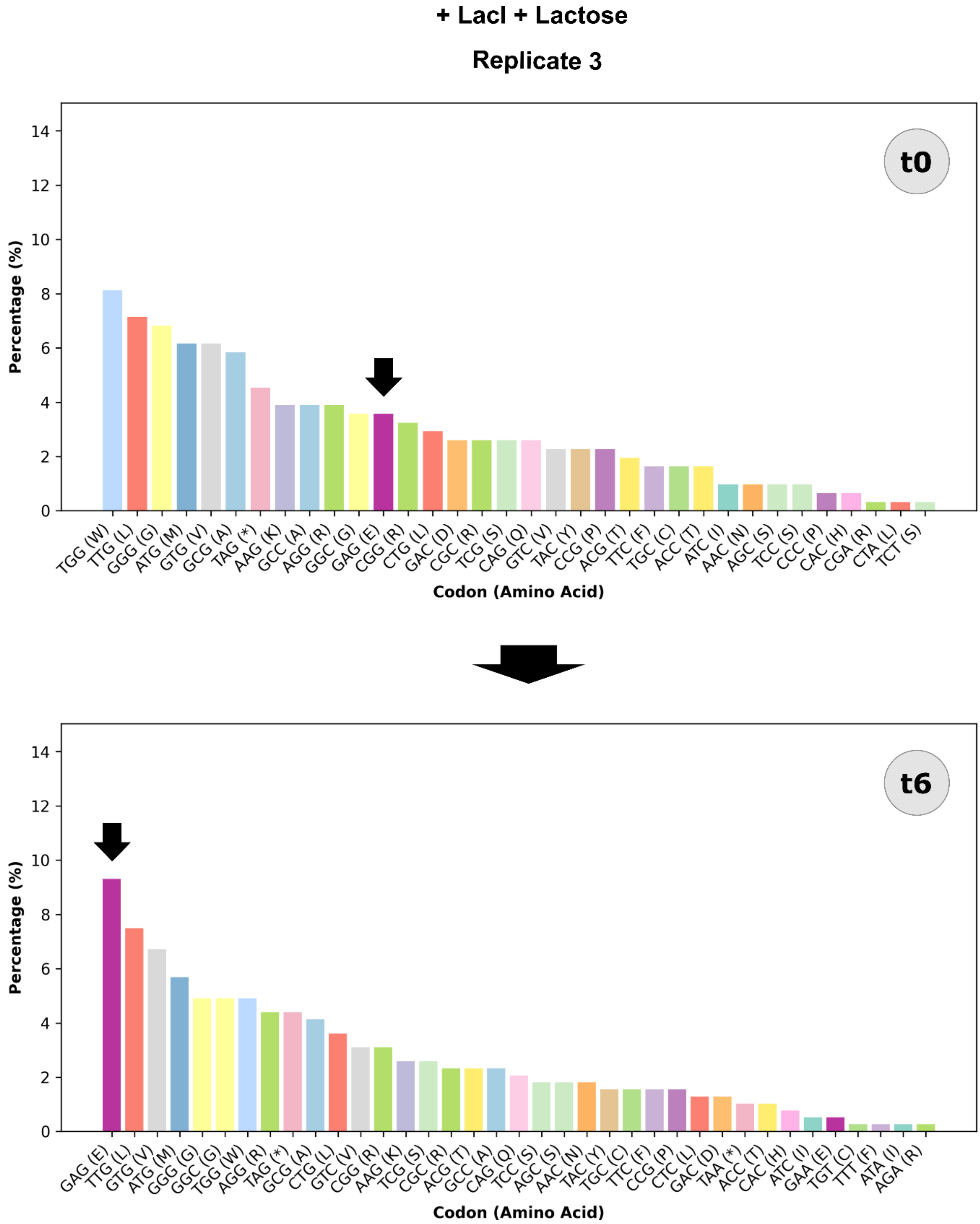

Fig. S12. Biological replicates of selection from a *lacZ* E538 codon library in the presence of LacI and lactose. The NNS-degenerate lacZ E538 codon library of ori-Op2Op3-lacZ was subjected to IVTTR in the presence of LacI and lactose. Nanopore FASTQ files were analyzed to determine the relative frequency of each codon encoding residue 538 before (t0) and after (t6) IVTTR. The GAG codon, encoding glutamate at position 538 (indicated with an arrow), was enriched across the three independent replicates. Codons encoding the same amino acid are represented with the same color. Supplementary Data is available for this figure.

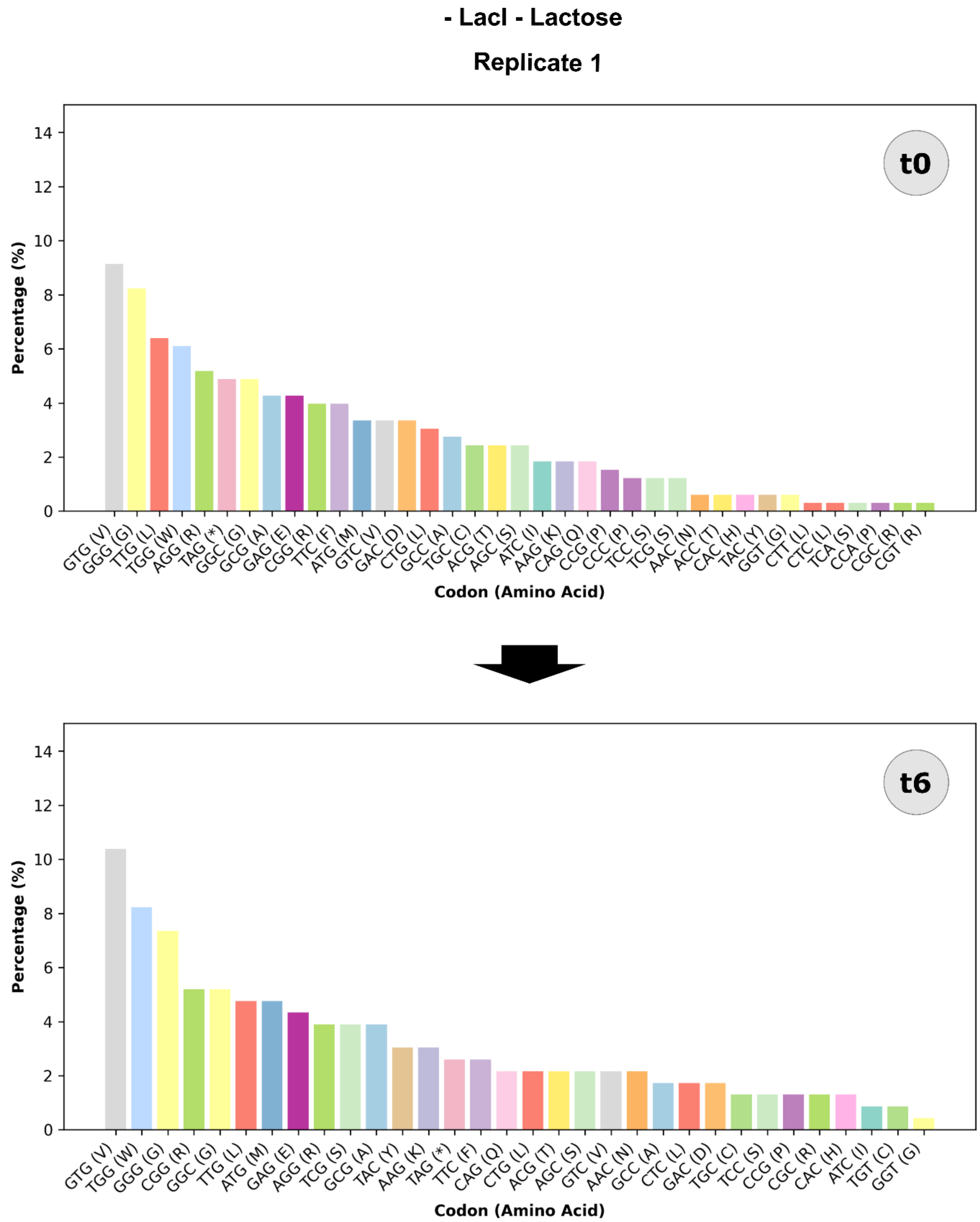

Fig. S13. *The figure continues on the next page*

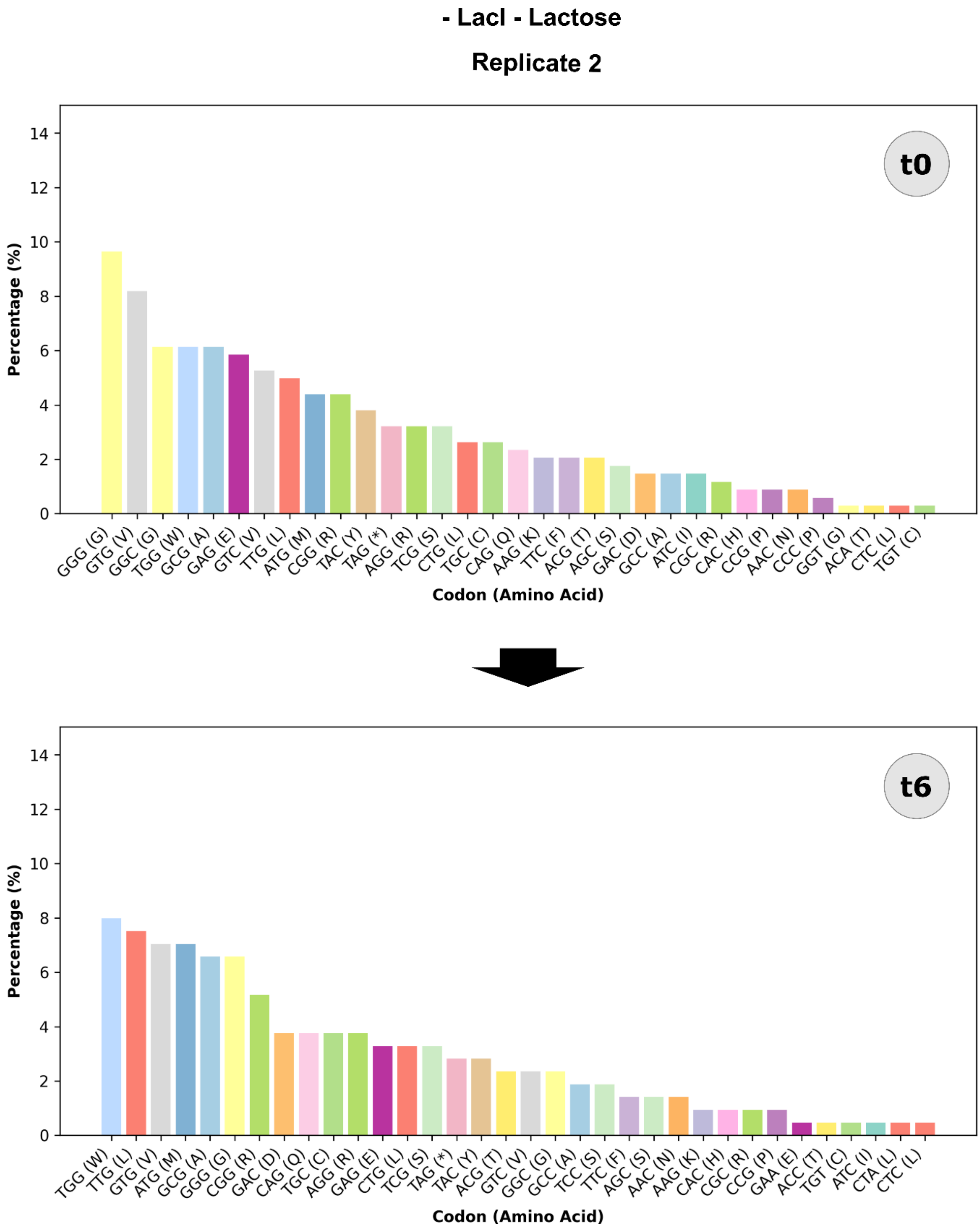

Fig. S13. *The figure continues on the next page.*

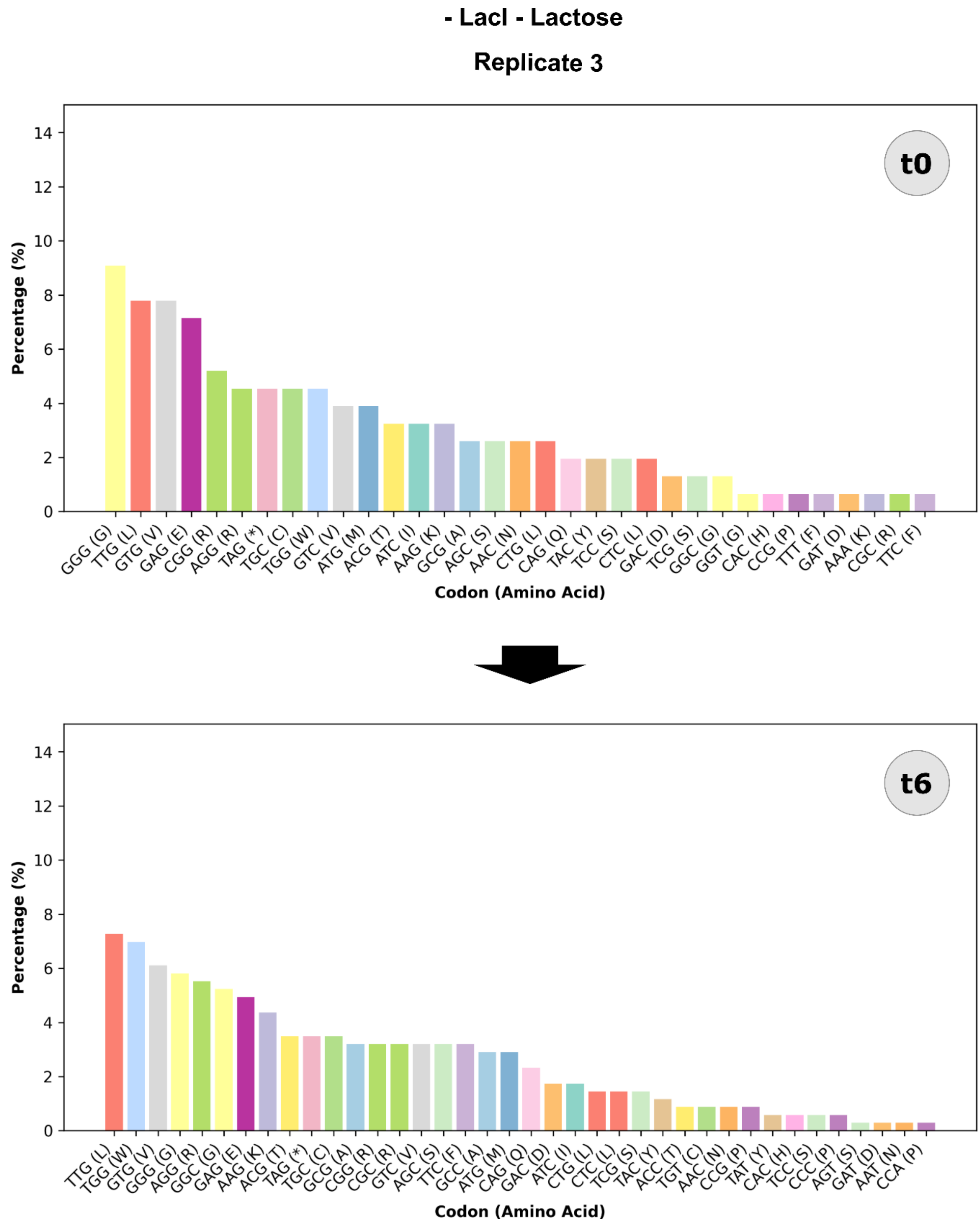

Fig. S13. Biological replicates of selection from a *lacZ* E538 codon library in the absence of LacI and lactose. The NNS-degenerate lacZ E538 codon library of ori-Op2Op3-lacZ was subjected to IVTTR in the absence of LacI and lactose. Nanopore FASTQ files were analyzed to determine the relative frequency of each codon encoding residue 538 before (t0) and after IVTTR (t6). No codons were selectively enriched. Codons encoding the same amino acid are represented with the same color. Supplementary Data is available for this figure.

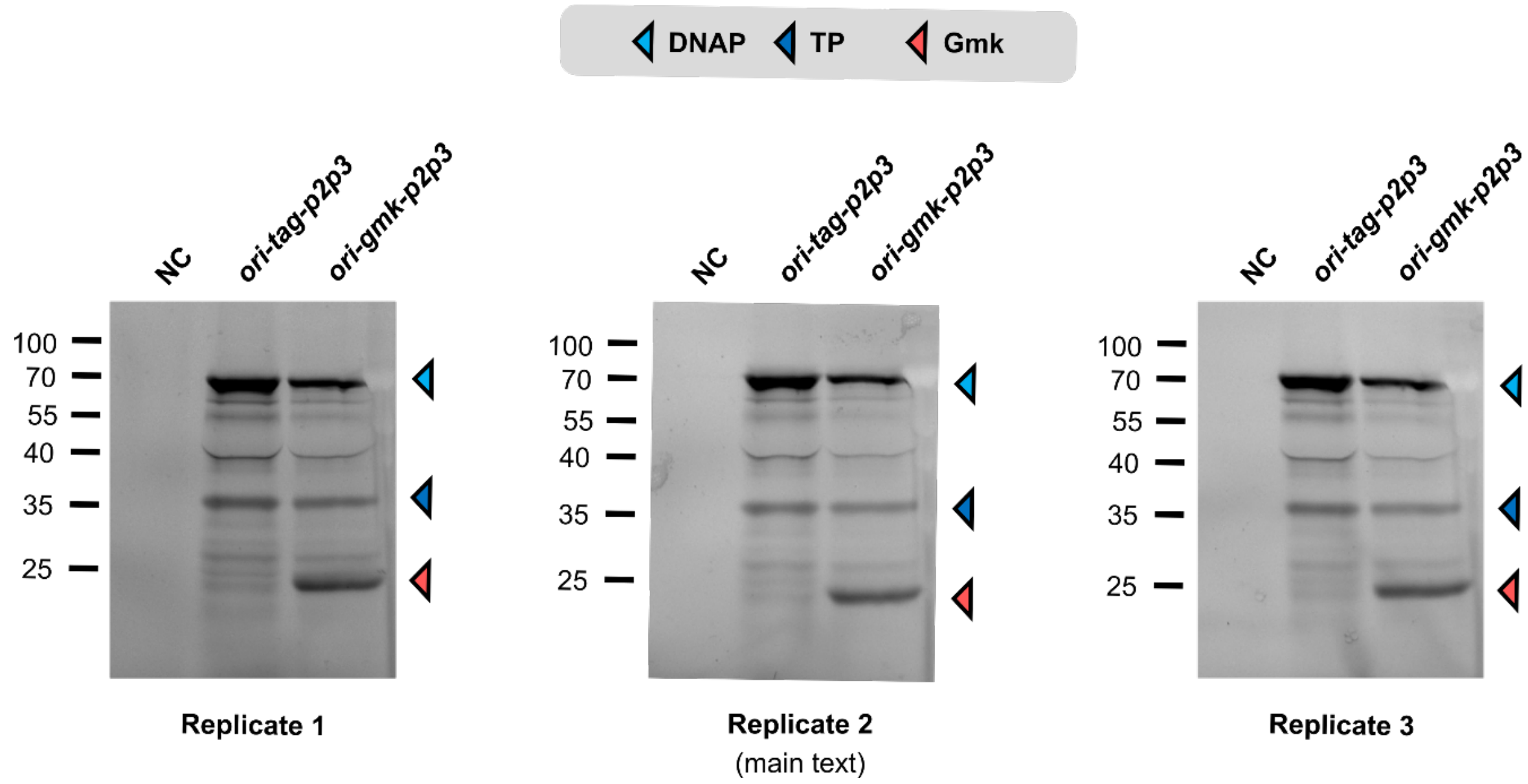

Fig. S14. Biological replicates of protein expression from *ori-tag-p2p3* or *ori-gmk-p2p3* DNA template. PURE reactions were supplemented with GreenLys reagent and visualized on SDS-PAGE gels. DNAP (~ 66 kDa), TP (~ 31 kDa), and Gmk (~ 24 kDa). NC: negative control with no DNA. Supplementary Data is available for this figure.
