# Supplementary material for: Autocatalytic selection of gene functions in synthetic cells
Source: Commun Biol. 2026 May 27;9:1124. doi: 10.1038/s42003-026-10372-z (PMC13493933; doi:10.1038/s42003-026-10372-z)
Supplement: Supplementary file 3 [file 42003_2026_10372_MOESM3_ESM.pdf]

Description of Additional Supplementary Files

File name: Supplementary Data
Description: Raw data including plotted data and uncropped gel scans and images are available within a zipped folder named 'Source Data'.
